# Mitochondrial Complex I Disruption Causes Broad Reorchestration of Plant Lipidome Including Chloroplast Lipids

**DOI:** 10.3390/ijms24010453

**Published:** 2022-12-27

**Authors:** Jean-Baptiste Domergue, Cinzia Bocca, Rosine De Paepe, Guy Lenaers, Anis M. Limami, Guillaume Tcherkez

**Affiliations:** 1 Institut de Recherche en Horticulture et Semences, Université d’Angers, INRAe, 42 Rue Georges Morel, 49070 Beaucouzé, France; 2 Unité Mitovasc, Institut de Biologie en Santé, Centre Hospitalo-Universitaire, Université d’Angers, 4 Rue Larrey, 49100 Angers, France; 3 Institute of Plant Science IPS2, Université Paris-Saclay, INRAe, Université Versailles-Saint-Quentin, Rue Noetzlin, 91450 Orsay, France; 4 Service de Neurologie, Centre Hospitalo-Universitaire d’Angers, 4 Rue Larrey, 49100 Angers, France; 5 Research School of Biology, College of Science, Australian National University, Canberra, ACT 2601, Australia

**Keywords:** mitochondrion, lipidomics, complex I, electron transfer chain, photorespiration, phospholipids, sphingolipids, cytoplasmic male sterility

## Abstract

Mitochondrial complex I (CI) plays a crucial role in oxidising NADH generated by the metabolism (including photorespiration) and thereby participates in the mitochondrial electron transfer chain feeding oxidative phosphorylation that generates ATP. However, CI mutations are not lethal in plants and cause moderate phenotypes, and therefore CI mutants are instrumental to examine consequences of mitochondrial homeostasis disturbance on plant cell metabolisms and signalling. To date, the consequences of CI disruption on the lipidome have not been examined. Yet, in principle, mitochondrial dysfunction should impact on lipid synthesis through chloroplasts (via changes in photorespiration, redox homeostasis, and N metabolism) and the endoplasmic reticulum (ER) (via perturbed mitochondrion–ER crosstalk). Here, we took advantage of lipidomics technology (by LC-MS), phospholipid quantitation by ^31^P-NMR, and total lipid quantitation to assess the impact of CI disruption on leaf, pollen, and seed lipids using three well-characterised CI mutants: CMSII in *N. sylvestris* and both *ndufs4* and *ndufs8* in Arabidopsis. Our results show multiple changes in cellular lipids, including galactolipids (chloroplastic), sphingolipids, and ceramides (synthesised by ER), suggesting that mitochondrial homeostasis is essential for the regulation of whole cellular lipidome via specific signalling pathways. In particular, the observed modifications in phospholipid and sphingolipid/ceramide molecular species suggest that CI activity controls phosphatidic acid-mediated signalling.

## 1. Introduction

Complex I (CI) is the first and largest of the four complexes of the mitochondrial electron transfer chain (mETC) [1]. It plays an important role in recycling NADH from catabolism (such as the tricarboxylic acid pathway and photorespiration) and participates in mitochondrial oxidative phosphorylation that generates cytoplasmic ATP. It is made of more than 40 subunits, most of which are nucleus-encoded except for a few ones encoded by the mitochondrial genome [2]. CI deficiency has been discovered and studied in many organisms from humans to plants. In humans, CI deficiency or dysfunction is responsible for mitochondrion-related diseases, such as LHON (Leber hereditary optic neuropathy), NARP (neurogenic muscle weakness, ataxia, and retinitis pigmentosa), MELAS (mitochondrial encephalomyopathy, lactic acidosis, and stroke-like episodes), or CPEO (chronic progressive external ophthalmoplegia), and has also been suggested to be involved in Parkinson’s disease [3]. In plants, many mutants carrying a CI deficiency have been characterised [4,5,6,7,8,9,10,11]. Because of alternative respiratory pathways, NAD(P)H dehydrogenases, and alternative oxidase (AOX), CI deficiency is generally not lethal in plants but is often associated with growth retardation and metabolic defects and occasionally morphologic abnormalities and male sterility (reviewed in [12]). Therefore, CI mutants are extremely useful for plant ecophysiology to examine the effects of the disturbance of mitochondrial homeostasis on redox power, photosynthesis, and N and C metabolism. 

To date, the best-characterised plant CI mutant is CMSII [13] in forest tobacco (*Nicotiana sylvestris* Speg. and Comes). The mitochondrial CMSII genome carries a deletion due to a recombination event affecting the region encoding the complex I NAD7 subunit [14]. CMSII plants display slow germination and growth, leaf abnormalities, and are partially male-sterile under high light with less than 50% of pollen able to germinate [14,15]. In CMSII mutants, their leaves show a general increase in antioxidative metabolism, a higher nitrogen-to-carbon ratio, and exhibit better resistance to abiotic and biotic stresses [16,17]. The higher relative N content is associated with an upregulation of chloroplastic N metabolism, in particular, amino acid synthesis. The slow growth phenotype has been linked to lower photosynthetic activity [18], which could be alleviated by elevated CO_2_ [19]. This effect is primarily caused by low Rubisco activation and low mesophyll conductance leading to lower CO_2_ concentration at carboxylation sites, which in turn leads to a high photorespiration rate [19,20]. 

Other well-characterised CI mutations affect nuclear genes, e.g., in *N. sylvestris,* NMS1 (*nuclear male sterility 1*) which is nearly completely male sterile and affected in mitochondrial gene splicing [21] and also *ndufs4* [22] and *ndufs8* [12] Arabidopsis mutants (*ndufs4* refers to the knockout mutant of the nuclear gene encoding fragment S subunit four; *ndufs8* refers to the knockout *ndufs8.1 ndufs8.2* double mutant of both nuclear genes encoded fragment S subunit eight). In Arabidopsis, the lack of subunits Ndufs4 and Ndufs8 prevent proper assembly of the holoenzyme. In contrast to *N. sylvestris* CI mutants, Arabidopsis *ndufs* mutants do not have a clear phenotype at the adult stage, and their pollen is fully fertile despite reduced seed germination and growth rates [10,12]. Unlike the CMSII mutant, these effects on growth in Arabidopsis *ndufs8* are due to slightly lower stomatal conductance rather than lower mesophyll conductance, causing lower photosynthesis and higher photorespiration [12].

Despite their differences, *N. sylvestris* and Arabidopsis mutants have similar metabolic alterations, and in particular, they are enriched in serine, which is mostly produced by photorespiration and is at the origin of the polar heads of phospholipids (serine, ethanolamine, and choline) and ceramides via sphingosine metabolism. GC-MS metabolomics analyses have shown that several precursors of lipid synthesis are amongst metabolites with a significant increase in CI mutants: digalactosyl-glycerol, glycerol, galactose, inositol, ethanolamine, tetradecanoate, and decanoate [12,23]. In addition, ^13^C-tracing with ^13^C-pyruvate in CMSII and NMS1 has shown differences in acetyl-CoA generation and utilisation compared to the wild-type, suggesting an increase in fatty acid synthesis [23]. Therefore, CI disruption seems to have important consequences on lipid metabolism and potentially could impact the balance between galactolipids, sphingolipids, and phospholipids. In human cells, respiratory defects lead to a modification of lipid composition not only in the mitochondrion but also in other cellular compartments. In cells carrying a mutation in *PINK1* (a gene responsible for inherited Parkinson’s disease) causing inefficient electron transfer between complex I and ubiquinone, there is an increase in ceramides [24,25]. In addition, the inhibition of fatty acid synthase (FASN2), which lowers palmitate and increases cardiolipins, has been shown to rescue the *pink1* phenotype [24]. Artificial generation of reactive oxygen species by xanthine oxidase triggers a concurrent decline of CI activity and cardiolipin content in mitochondria assayed in vitro [26]. In fibroblasts from LHON patients and thus with decreased CI activity, NMR-based metabolomics revealed a general increase in polyunsaturated fatty acids and phosphatidylcholine [27]. In many neurodegenerative disorders caused by mitochondrial dysfunction, an alteration of sphingolipid metabolism has been observed [28,29]. Interestingly, proteins involved in physical attachment and crosstalk between mitochondria and endoplasmic reticulum (ER), inositol 1,4,5-trisphosphate receptor 3 (IP3R), and voltage-dependent anion channel 1 (VDAC1), have been found to be impacted in fibroblasts from patients with CI deficiency [30]. This effect on ER can be of importance since it is the site of sphingolipid and ceramide synthesis.

In plants, the consequences of respiratory CI disruption on the lipidome have not been examined yet. Plant-specific consequences beyond mitochondrial membranes could be anticipated due to (*i*) alterations in photorespiration, redox homeostasis, and N metabolism that must impact chloroplast lipid synthesis and (*ii*) a possible alteration in mitochondrion–ER crosstalk. Here, we took advantage of lipidomics technology (by LC-MS), quantitation by ^31^P-NMR of phospholipids, and total lipid quantitation to assess the impact of CI disruption on the leaf, pollen, and seed lipids using three well-characterised CI mutants: CMSII in *N. sylvestris* and both *ndufs4* and *ndufs8* in Arabidopsis. Our results show multiple changes in cellular lipids, including galactolipids from the chloroplast and sphingolipids and ceramides usually synthesised by the ER, suggesting that mitochondrial homeostasis is essential for the regulation of cellular lipidome via specific signalling pathways between cell compartments.

## 2. Results

### 2.1. Overall Lipid Properties

**Figure 1:**
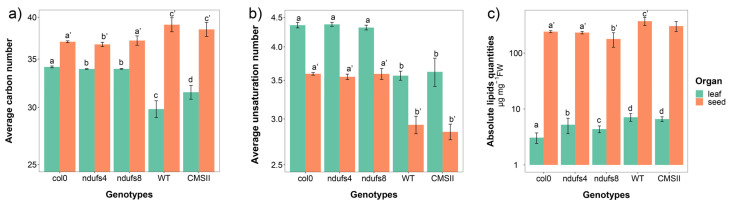
Chain length and unsaturation number in esterified fatty acid chains and absolute lipid content in leaves and seeds in wild-type and CI mutants CMSII (tobacco) and *ndufs4* and *ndufs8* (Arabidopsis). (**a**) Average chain length (sum of the two esterified fatty acids) across all lipid families; (**b**) average unsaturation number in esterified fatty acids number across all lipid families; (**c**) absolute content in total lipid (µg mg^−1^ FW). Letters stand for statistical classes (one-way ANOVA) both in leaves (nonprimed letters) and seeds (primed letters).

The overall average carbon and unsaturation number of fatty acid chains across all lipid families are shown in Figure 1. In leaves, fatty acid chains were found to be significantly shorter in *ndufs4* and *ndufs8* compared to the wild-type and longer in CMSII. In seeds, only *ndufs4* had significantly shorter chains (Figure 1a). No significant differences were found in average unsaturation between genotypes in both plant species (Figure 1b). The total extractible lipid content was also quantified and is shown in Figure 1c. In Arabidopsis leaves, both mutants *ndufs4* and *ndufs8* had significantly higher lipid contents than the wild-type. There was no significant difference in lipid content between CMSII and the wild-type in tobacco. The total extractible lipids included lipophilic pigments, and we found a slight increase in chlorophyll content in CMSII compared to the wild-type but no significant change in Arabidopsis mutants. In seeds, there was a significant reduction in total lipids in *ndufs8* only, likely due to the decrease in major, short-chain triglycerides (see below).

### 2.2. Leaf Lipidome

**Figure 2:**
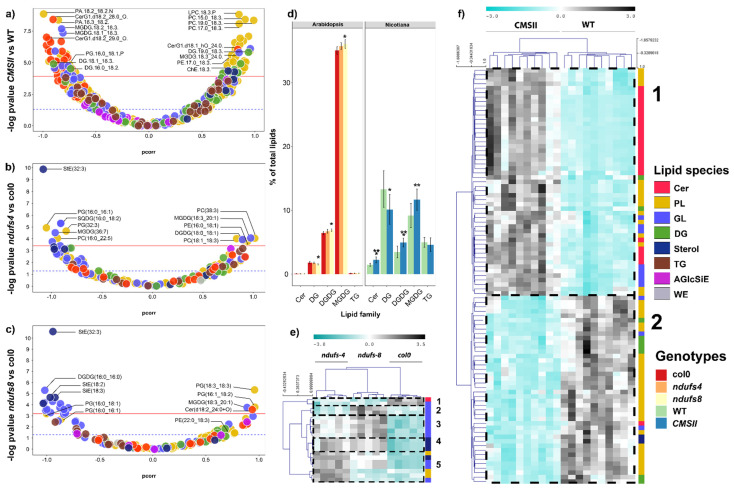
Leaf lipidome from LC-MS analysis in wild-type and CI mutants: CMSII (tobacco) and *ndufs4* and *ndufs8* (Arabidopsis). (**a**–**c**) Volcano plots showing the weight in sample discrimination (p_corr_ value from OPLS-DA multivariate analysis) against the *p*-value (Welch t-test, univariate analysis) to show best lipid biomarkers between mutants (on the left) and wild-type (on the right). (**d**) Relative LC-MS signal of whole lipid families in percentage of total lipid signal. (**e**,**f**) Heat map and hierarchical clustering (left) showing lipids that differed significantly (*p* < 0.05 with Bonferroni correction, two-way ANOVA) between genotypes. The colour scale stands for relative metabolite content from low (cyan) to high (grey, colour scale on top). The colour legend on the righthand side of this figure indicates lipid species families presented in (**a**–**c**) and genotypes in (**d**). A magnified version of this figure is provided in Supplementary Materials. Asterisks refer to statistical significance (*, *p* < 0.05; **, *p* < 0.01; ***, *p* < 0.001).

Lipidomics analyses allowed for the identification and quantitation of 417 lipid species in *Nicotiana sylvestris* and 155 in Arabidopsis leaves (Tables S1–S3). Multivariate analyses led to facile discrimination between sample types (genotypes) regardless of the plant species with Q² values of 0.716 for *ndufs4*, 0.771 for *ndufs8,* and 0.625 for CMSII; R² values of 0.969 for *ndufs4*, 0.962 for *ndufs8,* and 0.928 for CMSII; and *P*_CV-ANOVA_ was always lower than 0.0314. All three complex I mutants (CMSII, *ndufs4*, *ndufs8*) showed clear changes in lipidome compared with wild-type plants, with contrasting effects on lipid families (Figure 2). The volcano plots (Figure 2a–c) show the best lipid markers of genotypes, with mutants on the left (negative p_corr_ values) and wild-type on the right (positive p_corr_ values). In tobacco, CMSII leaves were enriched in many ceramides (red dots, Figure 2a) and depleted in many phospholipids (mostly phosphatidyl-choline (PC and LPC) species, yellow, Figure 2a) or some sterols (dark blue, Figure 2a). Specific glycoglycerolipids originating from the chloroplast and monogalactosyl diacylglycerols (MGDG, carrying C_18_ chains) were also found to be more abundant in CMSII. In Arabidopsis, both mutants appeared to have more glycoglycerolipids and phospholipids, with phosphatidylglycerols (PG) or digalactosyldiacylglycerols (DGDG) being most represented. Mutants also had more stigmasterol esters (dark blue, StE) (Figure 2b,c) and were depleted in various other lipids, including some specific phosphatidyl-choline species (PC) and ceramides. 

In terms of the prevalence of lipid families in total LC-MS ion signal, differences between genotypes were small in Arabidopsis with slightly fewer diacylglycerols and slightly more MGDG or DGDG (Figure 2d). This pattern was also found in tobacco with, again, an effect on ceramides, which was not visible in Arabidopsis due to the very small ceramide content in this species. Significantly different (i.e., *p*-value from ANOVA < Bonferroni threshold) lipid molecules were then grouped together in heatmaps with hierarchical clustering (Figure 2e,f). Similarly, genotypes could be easily classified with no error (tree on top of Figure 2f). There were relatively few significant lipids in Arabidopsis, and five covariation groups could be identified: As shown by groups two to five, mutants appeared to be enriched in several glycoglycerolipids, phospholipids, or sterols, in accordance with the volcano plot (Figure 2b,c). In tobacco, two big groups were visible, reflecting ceramides prevailed in more abundant lipids in CMSII, and phospholipids prevailed in more abundant lipids in the wild-type (Figure 2f). At this stage, there was no clear pattern in the molecular species of fatty acid esterification in lipids that were significantly different in mutants (Tables S1–S3; however, see below for unsaturation and chain length).

### 2.3. Seed Lipidome

**Figure 3:**
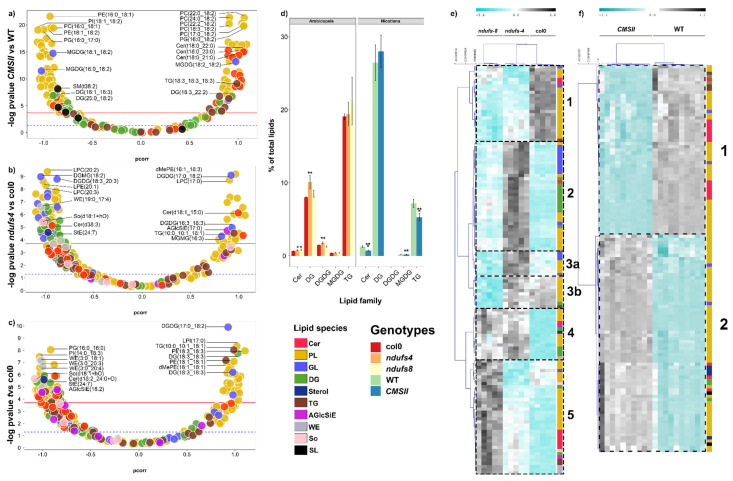
Seed lipidome from LC-MS analysis in wild-type and CI mutants: CMSII (tobacco) and *ndufs4* and *ndufs8* (Arabidopsis). (**a**–**c**) Volcano plots showing the weight in sample discrimination (p_corr_ value from OPLS-DA multivariate analysis) against the *p*-value (Welch t-test, univariate analysis) to show the best lipid biomarkers between mutants (on the left) and wild-type (on the right). (**d**) Relative LC-MS signal of whole lipid families in the percentage of total lipid signal. (**e**,**f**) Heat map and hierarchical clustering (left) showing lipids that differed significantly (*p* < 0.05 with Bonferroni correction, two-way ANOVA) between genotypes. The colour scale stands for relative metabolite content from low (cyan) to high (grey, colour scale on top). The colour legend at the bottom of this figure indicates lipid species families presented in (**a**–**c**) and genotypes in (**d**). A magnified version of this figure is provided in Supplementary Materials. Asterisks refer to statistical significance (*, *p* < 0.05; **, *p* < 0.01; ***, *p* < 0.001).

Seed lipidomics analyses led to the identification and quantification of 225 lipid molecules in *Nicotiana sylvestris* and 275 in Arabidopsis (Tables S4–S6). Many lipids were found to be significantly different between genotypes, allowing clear sample discrimination by multivariate analysis (Figure 3) with Q² values of 0.824 for *ndufs4*, 0.763 for *ndufs8,* and 0.891 for CMSII, respectively; R² values of 0.989 for *ndufs4*, 0.985 for *ndufs8*, and 0.995 for CMSII, respectively; and *P*_CV-ANOVA_ always lower than 4 × 10^−5^. In both plant species, several specific phospholipids species were differentially abundant between mutants and wild-type (Figure 3a–c). For example, some phosphatidyl-choline species (in particular, with a high C number in fatty acid chains) were more abundant in mutants while others were more abundant in the wild-type. In Arabidopsis, seeds of both mutants were enriched in diglycerides and ceramides, and in *ndufs4*, seeds had more glycoglycerolipids, wax esters, and sphingosine (Figure 3b,c). In terms of the LC-MS total signal of whole lipid families, the most visible differences in Arabidopsis were found in *ndufs4*, with significantly more diglycerides and DGDG. Triglycerides were too variable in the LC-MS total signal and therefore were not associated with significant differences between genotypes. In tobacco, CMSII seeds had fewer ceramides, MGDG, and triglycerides (Figure 3d). When represented as a heatmap with hierarchical clustering, significantly different lipid species were numerous and formed five to six groups in Arabidopsis. Each group comprised lipids of different families, showing that changes in the regulation of lipid synthesis or degradation were not influencing whole lipid families but affected specific lipid species. This pattern was also found in tobacco. It is also worth noting that the two Arabidopsis mutants did not behave similarly, with differences in lipid species affected by the mutation. In fact, unlike *ndufs4*, *ndufs8* seeds appeared to be depleted in glycoglycerolipids (GL) and enriched in acylglucosyl-sitosterol esters (AGlcSiE).

### 2.4. Pollen Lipidome in Tobacco

**Figure 4:**
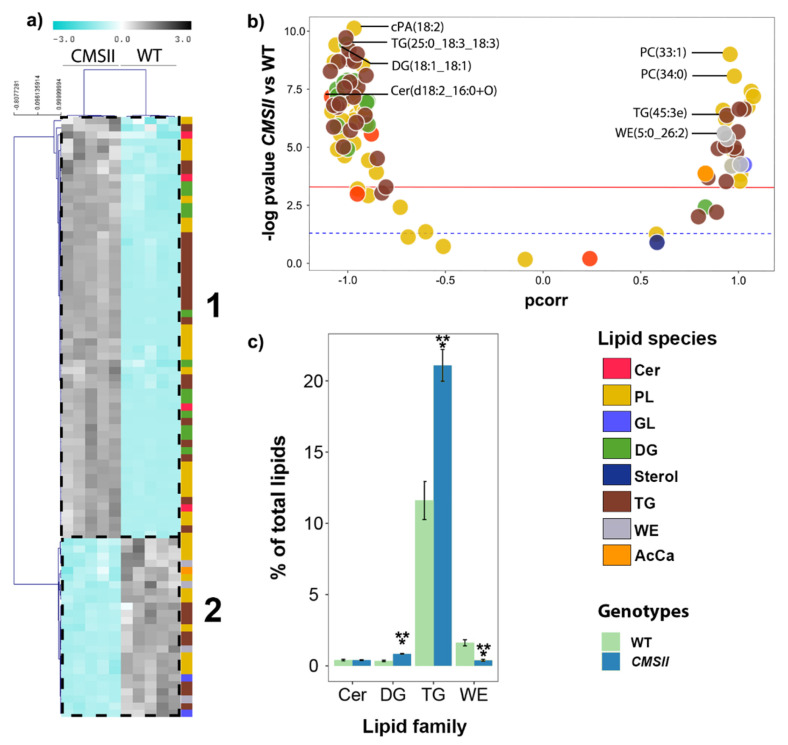
Pollen lipidome in tobacco from LC-MS analysis in wild-type and CMSII. (**a**) Heat map and hierarchical clustering (left) showing lipids that differ significantly (*p* < 0.05, two-way ANOVA) between genotypes. Colour scale as in Figure 1 and Figure 2. (**b**) Volcano plot showing the weight in sample discrimination (p_corr_ value from OPLS-DA multivariate analysis) against the *p*-value (Welch t-test, univariate analysis) to show best lipid biomarkers between CMSII (on the left) and wild-type (on the right) pollen. (**c**) Relative quantity of lipid families of major importance in percentages of total signal. The colour legend on the right-hand side indicates lipid species families presented in the volcano plots. Genotypes are indicated with light green (wild-type) and dark turquoise (CMSII) in (**c**). A magnified version of this figure is provided in Supplementary Materials. Asterisks refer to statistical significance (***, *p* < 0.001).

Lipidomics analyses could only be performed in tobacco since the pollen quantities obtained in Arabidopsis were insufficient to allow for proper quantification. The results are shown as a heatmap (Figure 4a) and volcano plot (Figure 4b). CMSII pollen was enriched in triglycerides (including triglycerides esterified with long-chain fatty acids) along with some diglycerides (DG) and a few ceramides (Figure 4a,b). On the other hand, CMSII pollen was depleted in wax esters. Differences in triglycerides were also visible in the total LC-MS ion signal of lipid families (Figure 4c), with nearly a two-fold increase in triglycerides in CMSII pollen.

### 2.5. Leaf and Seed Phospholipids

**Figure 5:**
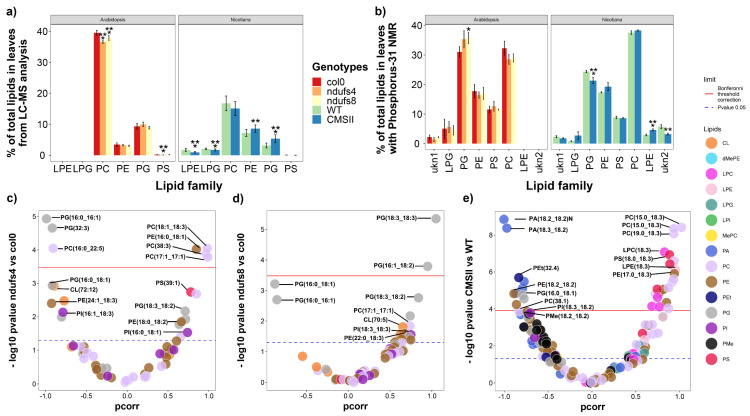
Leaf phospholipid pattern from both LC-MS and ^31^P NMR analysis in wild-type and CI mutants: CMSII (tobacco) and *ndufs4* and *ndufs8* (Arabidopsis). (**a**) Phospholipid families’ contents in percentage of total LC-MS signal. (**b**) Phospholipid families in percentage of total absolute phospholipid amount determined by ^31^P-NMR. (**c**–**e**) Volcano plots showing the weight in sample discrimination (p_corr_ value from OPLS-DA multivariate analysis) against the *p*-value (Welch t-test, univariate analysis) to show best lipid biomarkers between mutants (on the left) and wild-type (on the right). The colour legend on right indicates phospholipid subfamilies in (**c**–**e**). A magnified version of this figure is provided in Supplementary Materials. Asterisks refer to statistical significance (*, *p* < 0.05; **, *p* < 0.01; ***, *p* < 0.001).

**Figure 6:**
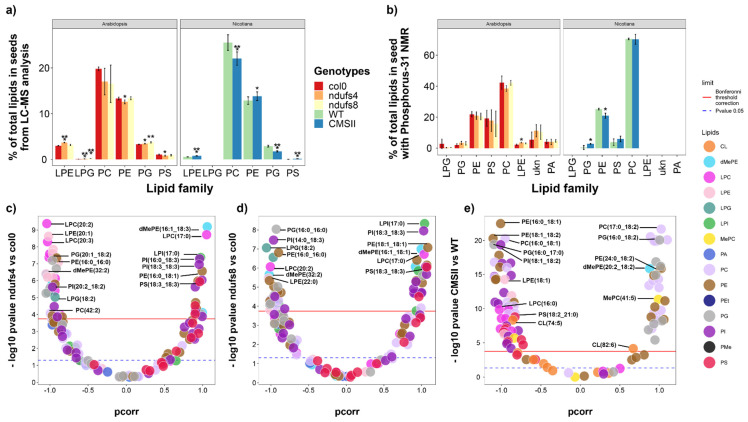
Seed phospholipid pattern from both LC-MS and ^31^P NMR analysis in wild-type and CI mutants: CMSII (tobacco) and *ndufs4* and *ndufs8* (Arabidopsis). (**a**) Phospholipid families’ contents in percentage of total LC-MS signal. (**b**) Phospholipid families in percentage of total absolute phospholipid amount determined by ^31^P-NMR. (**c**–**e**) Volcano plots showing the weight in sample discrimination (p_corr_ value from OPLS-DA multivariate analysis) against the *p*-value (Welch t-test, univariate analysis) to show best lipid biomarkers between mutants (on the left) and wild-type (on the right). The colour legend on right indicates phospholipid subfamilies in (**c**–**e**). A magnified version of this figure is provided in Supplementary Materials. Asterisks refer to statistical significance (*, *p* < 0.05; **, *p* < 0.01; ***, *p* < 0.001).

A specific analysis of phospholipid composition was conducted using both LC-MS (relative signals) and ^31^P-NMR (absolute contents) in both the leaves (Figure 5) and seeds (Figure 6). The use of both techniques is extremely useful to appreciate concurrent changes in quantity and chemical composition. The effect of CI mutation was not similar in the two plant species (Figure 5a,b), which is not surprising considering their huge difference in leaf phospholipid composition.

In CMSII leaves, lipidomics data indicate that lyso forms (i.e., with only one fatty acid chain esterified onto the glycerol backbone) were less abundant while phosphatidyl-ethanolamine and phosphatidyl-glycerol were more abundant (Figure 5a). NMR absolute quantification also suggested a change in lyso-phosphatidyl-ethanolamine and phosphatidyl-glycerol but in the opposite direction (Figure 5b). This difference between techniques is not surprising because LC-MS-based lipidomics are influenced by the ionisation efficiency of lipid species. Therefore, if changes in the contents are accompanied by changes in composition and thus in ionisation efficiency within a lipid family, the total LC-MS signal of a lipid family may not reflect its effective content. By contrast, NMR analysis provides a direct quantitation of phosphorus atoms. This difference is also visible when comparing phospholipid classes. For example, phosphatidyl-serine (PS) are less easily ionisable and represented a very small signal in LC-MS (≈ 1%, Figure 5a) whereas it represented nearly 10% of phospholipids in absolute terms (Figure 5b). CMSII was depleted in several forms of phosphatidyl-ethanolamine esterified with an 18:3 fatty acid chain (for example, linolenic) but enriched in forms with an 18:2 chain (for example, linoleic) (Figure 5e, Table S3). Moreover, despite the lack of change in total phosphatidyl-choline amount (Figure 5a,b), there was a change in molecular species composition, with more species esterified with an 18:3 fatty acid chain (Figure 5e). Although at a lower significance level, there was a general increase in phosphatidyl-methanol (PMe) and a decrease in phosphatidyl-serine (PS) in CMSII (black and red dots, Figure 5e).

In Arabidopsis, there was a significant decrease in phosphatidyl-choline in both mutants and no change in phosphatidyl-glycerol (PG) in the LC-MS signal accompanied by an increase in absolute content in *ndufs8* (Figure 5a,b). This effect was caused by the prevalence of PG molecular species less prone to ionisation typically carrying shorter and/or less unsaturated fatty acid chains, such as PG(16:0,16:1) at the expense of PG(18:3,18:3) (Figure 5c). Interestingly, we also noticed a change in cardiolipin CL(72:12) (increased) and phosphatidyl-serine PS(39:1) (decreased) (Figure 5c). 

In seeds, there were rather subtle and similar changes in the LC-MS signals of the phospholipid families in the mutants of both plant species. Lyso-phosphatidyl-ethanolamine (LPE) increased while phosphatidyl-choline (PC) decreased on average, and there was a change in phosphatidyl-ethanolamine (PE) and phosphatidyl-glycerol (PG) (Figure 6a). In terms of absolute content measured by ^31^P-NMR, most changes were insignificant in Arabidopsis (except for an increase in LPE in *ndufs4*). In tobacco, CMSII seeds had more PG and less PE (Figure 6b). Many molecular species of phosphatidyl-serine were less abundant in Arabidopsis mutants (red dots) while CMSII was enriched in many molecular species of phosphatidyl-inositol (purple dots) (Figure 6c–e).

## 3. Discussion

### 3.1. Pollen Lipid Composition and Possible Relationship with Partial Male Sterility

The most striking effects of complex I mutation in CMSII were the increase in triglycerides (TG) and also diglycerides (DG) to a lower extent (Figure 4), in particular, TG with fatty acids carrying three degrees of unsaturation (linolenic type) (Table S7). Despite the concurrent increase in some DG species, it is possible that the general TG increase reflects a change in the activity of DG acyl transferase, which catalyses the last step of TG synthesis [31]. It is also worth noting that DG are precursors of phosphatidic acid (PA) via diacylglycerol kinase (DGK), and PA is a second messenger that can affect developmental signalling. Here, it is possible that DG accumulation in mutants translates into lower total PA concentration and thus weaker signal for pollen tubes growth signal. As such, partial male sterility in CMSII could be the consequence of a change in DGK activity, which has been linked to tip growth regulation [32] via the PA signal [33]. Unfortunately, the PA content in pollen was too small and only two molecular species of PA could be quantified using LC-MS. Appropriate phospholipid concentration is required to control pollen tube growth [34]. Here too, PA signalling could be involved since it can be produced by phospholipase D from phospholipid cleavage. In our analyses, most phospholipids found in pollen were phosphatidyl-choline, and CMSII pollen was enriched in esterified forms with long fatty acid chains (38 C-atoms and above) (Table S7). In terms of carbon balance, the build-up of TG in CMSII pollen probably took place at the expense of other compounds participating in pollen maturation and/or pollen tube development, such as soluble sugars and starch, which are essential for pollen maturation [35,36].

### 3.2. Complex I Mutations Affect Chloroplast Lipids

Surprisingly, in leaves, the most visible effect of mitochondrial complex I mutation was on chloroplastic lipids (galactolipids) (Figure 2). Galactolipids (digalactosyl-diacylglycerol, DGDG, and monogalactosyl-diacylglycerol, MGDG) account for up to 75% of lipids in chloroplast envelopes [37] and form two major lipids families of the thylakoid membrane [38]. Here, we found that DGDG/MGDG relative quantities were higher in all mutants and carried longer carbon chains and more unsaturation in both Arabidopsis and tobacco. It is likely that these modifications have consequences on the physical properties of chloroplast membranes [38,39], such as membrane fluidity [38], and this might contribute to higher resistance to PSII photoinhibition [40,41,42]. Moreover, the MGDG/DGDG ratio is critical for the appropriate structure of thylakoid membranes including interactions with membrane proteins of the photosynthetic machinery [43]. In other words, the observed changes in the galactolipids should impact on photochemistry and contribute to explaining the lower photosynthetic activity observed in CMSII [20] but not in the *ndufs* mutants that have unaltered photosynthesis rates at a fixed intercellular CO_2_ mole fraction. Moreover, this could contribute to better tolerance mechanisms in both the CMSII and *ndufs* mutants, which possess enhanced stress resistance [10,44].

The mechanisms responsible for the change in galactolipids in complex I mutants are presently uncertain. Metabolomics on rosette leaves has shown that *ndufs* mutants have more free sugars, including galactose, galactonate, *myo*-inositol, and raffinose [12], suggesting an upregulation of galactose/inositol biosynthesis. Moreover, it should be noted that galactolipid synthesis is primarily controlled by monogalactosyldiacylglycerol synthase 1 (MGD1), the activity of which increases under stress conditions, such as phosphorus deficiency and drought [45]. Moreover, MGD1 activity is stimulated by phosphatidyl-glycerol (PG) [46] and phosphatidic acid (PA), the latter playing a crucial role in galactolipid synthesis regulation [47]. In Arabidopsis, PA was at too low a concentration to be detected. In tobacco, nearly all PA molecular species were more abundant in CMSII (Figure 5e), suggesting that PA-based regulation of galactolipids is plausible.

### 3.3. Complex I Mutations Affect Phospholipids and Alter Ethanolamine Metabolism

A summary of the metabolic schematics is provided in Supplementary Figure S1. In leaves, Arabidopsis mutants presented a rise in PG at the expense of phosphatidyl-choline (PC), but none of these two phospholipid families is clearly segregated in the volcano plots (Figure 5c,d), meaning specific molecular species within families are affected rather than families as a whole. The tobacco mutant CMSII showed many more significant lipids (Figure 5e) with no change in PC content, perhaps because of homeostasis mechanisms since PC is the major membrane component. We also found a general increase in phosphatidyl-methanol (PMe) in CMSII leaves, which is a product formed by phospholipase D (PLD) via trans-phosphatidylation of phosphatidyl-choline and phosphatidyl-ethanolamine with short-chain alcohols during extraction [48]. Although PMes are probably not natural lipids present in membranes, their higher content in the lipidome reflects higher PLD activity in CMSII. It is worth noting that in vivo, PLD performs phospholipid cleavage, generating phosphatidic acid (PA). As mentioned above, many PA species were much more abundant in CMSII compared to the wild-type. Besides possible impacts of PA on galactolipids, PA build-up could also have, by mass balance, an effect on some diacylglycerol (DG) species and consequently on the TG which come from DG. PA is a precursor of DG [49], and, consequently, a lower consumption of PA could lead to lower DG and/or TG content, as observed in CMSII. This could be related to the downregulation of PA phosphatases, leading to PA accumulation and thus PA-mediated signalling [50]. Interestingly, CMSII leaves were found to be enriched in ceramides (Figure 2a), perhaps suggesting a link with leaf shape alterations and necrosis via ceramide-mediated enhancement of programmed cell death [51]. 

In Arabidopsis seeds, there was a clear stimulating effect of CI mutations on phosphatidyl-serine (PS) and ceramide content (Figure 3 and Figure 6), suggesting a general impact on the biochemical pathway of the whole family. In particular, both biosynthetic pathways use cytosolic serine and thus are in competition when serine concentration is low. Serine palmitoyl transferase (SPT) attaches palmitoyl-CoA to a serine as the committed step of the sphingolipid biosynthetic pathway, and phosphatidylserine synthase (PSS) attaches CDP-diacylglycerol to serine (as part of the CDP-DAG-dependent pathway). Importantly, other phospholipids of the CDP-DAG pathway were not affected, suggesting a specific upregulation of sphingolipid biosynthesis. In fact, SPT has been found to be stimulated by various stresses affecting the endoplasmic reticulum [52], resulting in the accumulation of ceramides (as observed in Figure 3). Moreover, other mechanisms could play a role, such as a downregulation of PSS or lower serine delivery from phloem sap during seed development. 

In tobacco, CMSII seeds were enriched in PS and phosphatidyl-inositol (PI) and depleted in PG, strongly suggesting that within the CDP-DAG pathway, there was a downregulation of the mitochondrial cardiolipin pathway (which synthesises both PG and cardiolipins), rerouting lipid production to PS and PI. Plant mitochondria are capable of cardiolipin and PG synthesis as demonstrated in mung beans [53]. In general, conditions that affect mitochondrial function impact mitochondrial lipid content, including cardiolipins and phosphatidyl-glycerol, and reciprocally, mitochondrial lipid composition impacts the respiratory electron transfer chain [54]. The first committed step of the cardiolipin pathway is phosphatidyl-glycerol phosphate synthase (PGS), which is inhibited by inositol via a post-translational phosphorylation modification [55]. Interestingly, inositol and derived inositides are part of a broad regulation network under stress conditions [56,57] and this might be involved in CMSII. It has also been shown in Arabidopsis that reciprocally, the alteration of cardiolipin synthesis (in the *cardiolipin synthase* mutant, *cls*) leads to mitochondrial defects [58].

## 4. Materials and Methods

### 4.1. Plant Material

Complex I mutants used here have already been characterised in two species of interest, forest tobacco (*N. sylvestris*) and Arabidopsis. *N. sylvestris* CMSII has a deletion in its mitochondrial genome as the result of a recombination event [59] and lacks the entire *nad7* gene encoding the NAD7 subunit of the membrane arm of Complex I [60]. The Arabidopsis *ndufs4* T-DNA insertion mutant (At5g67590) comes from the Syngenta collection [22] and lacks the Ndufs4 core subunit [10]. The Arabidopsis *ndufs8* T-DNA insertion mutant is the double mutant affected in both At1g16700 and At1g79010 genes (original *ndufs8.1* and *ndufs8.2* single mutants from the Salk Institute collection) and lacks the core Ndufs8 76 kDa subunit (Petriacq et al. 2017). In all mutants, the lack of the subunit impairs assembly of the holoenzyme. Seeds used to sow and grow plants were obtained from plants cultivated in a controlled temperature room under long days.

### 4.2. Plant Cultivation

Plants were sown directly in pots and grown in a controlled temperature room. CMSII mutant and wild-type (WT) tobacco were grown under long days (16 h light/8 h dark) at 230 µmol m^−2^ s^−1^ light intensity at constant temperature (19 °C). Plants were watered on a daily basis and supplemented with 2 g/L of Peters Professional^TM^ Pot Plant Special (NPK 15−11−29 + TE) once a week. After germination and emergence directly in pots, Arabidopsis seedlings were thinned, and plants were grown under short days (8 h light/16 h dark) at 145 µmol m^−2^ s^−1^ light intensity and constant temperature (19 °C) and supplemented with 1.5 g/L of Plant Prod^®^ (NPK 15−10−30).

### 4.3. Sampling

For both species, leaf harvesting was done just before floral stem elongation. Leaf samples (≈90 mg) were harvested, quickly weighted, immediately put in liquid nitrogen, and stored at −80 °C until analysis. In tobacco, only a small portion (without main veins) of leaves was harvested (given the large size of each leaf) while for Arabidopsis, the entire rosette was used. Roots were not sampled. Pollen from tobacco flowers was obtained from plants grown in the greenhouse to accommodate the large number of plants needed to produce enough pollen for experiments. For 15 days, pollen was collected from flowers by delicate shaking to retrieve a small amount of pollen falling off stamens. Pollen from Arabidopsis flowers was collected in plants grown under long days using an adapted vacuum cleaner comprising an HEPA filter to harvest pollen from ≈ 200 inflorescences.

### 4.4. Lipidomics Analyses

Sampled were first ground with 0.6 mL methanol in 2 mL Eppendorf tubes with two 5 mm inox beads using a Qiagen Tissue Lyser. Ground samples were transferred to a glass tube, 1 mL CDCl_3_ was added, and then 300 µL of KCl (0.88%). After centrifugation at 2000× *g* for 4 min, the organic phase was transferred into silanised vials and spin-dried. Analyses were then conducted as in [61]. Samples were then resuspended in acetonitrile–isopropanol–water (65/35/5, v/v/v) plus 10 µL internal standard (mixture of deuterated standards at 10 µL/mL each). Samples were analysed with an Ultimate 3000 ThermoFisher UHPLC coupled with Q-Exactive^TM^ mass spectrometer (ThermoScientific, Courtaboeuf, France). Injection of samples was randomised, and quality controls (QC) made of aliquots of all samples were also injected every 10 samples. The LC column was Phenomenex Kinetex 1.7u Evo-C18 100Å (150 × 2.1 mm) 45 °C nominal temperature. Data were analysed using LipidSearch^TM^. Files containing full-scan MS and data-dependent MS^2^ were searched for all known lipids using the software library. Results were then aligned and filtered out for misidentification and low signal-to-noise ratio. TraceFinder^TM^ was then used to compare manually each identified lipid in all samples one by one. All identified lipids in samples were compared to the same lipid in QC MS^2^ spectra with LipidSearch^TM^. Several criteria were used to select reliable lipids: (*i*) QC with CV (coefficient of variation) area below 30%; (*ii*) a perfect match of the isotopic pattern (compared to the simulated isotopic based on elemental composition) and an m/z variation below 10 ppm; (*iii*) linearity of dilution with an r² higher than 0.7; (*iv*) consistency for each lipid species for retention time (RT), the number of carbon (*N*), and unsaturation (*U*). To do this, each family of lipids was represented in a scatter plot (*RT* plotted against *N*), and a trend line was drawn according to unsaturation (*U*). Any lipid falling outside the trend line was considered a misidentification. Data were normalised using the MS total useful signal (MSTUS). Abbreviations associated with lipid molecular species are listed in List S1 in Supplementary Material. The full curated list of detected and quantified lipids is shown in Tables S1–S7 along with *p*-values and OPLS loadings (see *Statistics* below).

### 4.5. Phospholipid Analyses

Samples were ground with 1.2 mL methanol as above. Ground samples were transferred to a 15 mL Falcon^®^ tube, and 4 mL methyl-*t*-butyl ester (MTBE) was used to rinse the Eppendorf tube and poured in the Falcon^®^ tube which was then placed into a tube-shaker at 4 °C for 1 h. One mL MilliQ water was then added; tubes were shaken again for 10 min, centrifuged at 10.000× *g* for 10 min, and the organic phase was transferred to a glass tube. The sample was then re-extracted with 4.12 mL MTBE, shaken for 1 h, centrifuged at 10.000× *g*, and the organic phase was added to the first one. The extract was then spin-dried. Samples were resuspended in methanol/CDCl_3_ (with triphenyl phosphonate as an internal standard)/cholic acid solution in D_2_O (2/1/0.8, v/v/v). This formed only one phase that could be transferred into 5 mm NMR tubes. Samples were analysed using an Advance 500 MHz spectrometer (Bruker Biospin, Wissembourg, France) with the following parameters: lock on deuterium, pulse program zgpg, 90° impulsion, D1 0.4 s, AQ 0.4 s, proton uncoupling sequence waltz16, 32.000 scans. Data were analysed using TopSpin^TM^ 4.1.3. Peak areas were normalised using the internal standard and fresh weight.

### 4.6. Total Lipid Content

Samples were extracted with 4 mL hexane in 5 mL Eppendorf tubes and ground with four 5 mm inox beads using the Qiagen Tissue Lyser. One mL methanol was added; samples were vortexed, and 1 mL KCl (0.88%) was added. Samples were then centrifuged at 10.000× *g* and the organic phase was transferred to 2 mL Eppendorf tubes for evaporation under N_2_ flow. A total of 300 µL hexane was added to resuspend the lipid pellet and transferred to a preweighed tin capsule (Elementar, Villeurbanne, France). After hexane evaporation, capsules were weighted using a microbalance (Sartorius, Les Ulis, France).

### 4.7. Statistics

Five true replicates (individual plants) were used under all conditions. Supervised multivariate analysis of lipidomics data was carried out by orthogonal projection on latent structure (OPLS) with Simca^®^ 16 (Umetrics, Umeo, Sweden) using genotype as the Y response variable and lipids as predictive X variables. The absence of statistical outliers was first checked using principal component analysis (PCA) to verify that no data point was outside the 99% confidence Hotelling region. The goodness of the OPLS model was appreciated using the determination coefficient R², and the predictive power was quantified by the cross-validated determination coefficient, Q². The significance of the statistical OPLS model was tested using a χ² test that compares with a random model (average ± random error), and the associated *p*-value (*P*_CV-ANOVA_) was reported. A permutation test was also performed to check the reliability of the OPLS model, that is, to verify that at maximal permutation (similarity of permuted dataset tending to zero), Q², was always negative. Best discriminating lipids (lipid biomarkers) were identified using volcano plots whereby the logarithm of the *p*-value obtained in univariate analysis was plotted against the rescaled loading (p_corr_) obtained in the OPLS. In such a representation, best lipid biomarkers have both maximal –log(*P*) and p_corr_ values. Univariate analysis was performed using Student–Welch T-test (pairwise) or ANOVA (two classes or more) with a threshold of *p* = 0.05 or adjusted for false discovery rate using the Bonferroni correction, as indicated in figures.

### 4.8. Calculations

Carbon numbers and unsaturation of fatty acid chains were calculated in Excel^®^ using weighted averages via the instruction *sumprod* whereby the vector of number of carbons was multiplied by the vector of normalised areas. Here, normalisation used total ion signal, i.e., the sum of all integrated areas of all lipids retained, in the analysis. The same method was used for unsaturation except that the number of unsaturation was used instead of the number of carbon atoms.

## 5. Conclusions

Leaf, pollen, and seed lipidomes show alterations caused by mitochondrial complex I disruption. Although different mechanisms are involved in Arabidopsis and tobacco and in different tissues, mitochondrial dysfunction leads to lipid reorganisation, including in plastids. In leaves, changes in primary carbon metabolism, such as increased *myo*-inositol and raffinose metabolisms as well as increased cellular stress, in particular, via PA liberation, MDG, and PSS activities, triggered by complex I disruption, represent good candidates to explain changes in galactolipids and phospholipids. PA is generated by either phospholipase D (PLD) which cleaves phosphatidyl-choline and phosphatidyl-ethanolamine, diacylglycerol kinase (DGK) via diacylglycerol phosphorylation, or phospholipase C (PLC) which cleaves phosphatidyl-inositol [62]. Further work is needed to quantify PA precisely, assay associated enzymatic activities, and thus decipher PA origin in mutants. Interestingly, changes in the lipidome appear to be more complicated than just a general increase in serine-consuming pathways (e.g., increase in PS, PE, and PC content), as our previous metabolomics and physiological data had suggested (due to increased photorespiration, at least in CMSII, see *Introduction*). Nevertheless, observed changes in lipids are probably linked to stress responses, such as oxidative stress generated by both photorespiration and mitochondrial dysfunction. For example, PLC activity is stimulated during endoplasmic reticulum stress response [63], and a meta-analysis has revealed that galactolipids (and the MDGD/DGDG ratio) are modulated under a variety of abiotic stresses, including drought, which increases photorespiration [64]. In addition, there is a correlation between galactolipid synthesis regulation (expression of genes encoding for MGD1) and the expression of genes associated with the photorespiratory pathway (in peroxisomes and mitochondria) and the glyoxylate cycle [65]. Moreover, galactolipid synthesis (MGD1 activity) is modulated by the redox power in the chloroplast [66]. Further work is warranted to gain insights into the dynamics of lipid synthesis (using, e.g., isotopic labelling) and generate a full picture of regulated steps in lipid metabolism (using proteomics analyses). This will be addressed in a subsequent study.

## Data Availability

Data supporting reported results can be found in the Supplementary Materials.

## Supplementary Materials

The following supporting information can be downloaded at https://www.mdpi.com/article/10.3390/ijms24010453/s1.
