# Supplementary material for: Mitochondrial Complex I Disruption Causes Broad Reorchestration of Plant Lipidome Including Chloroplast Lipids
Source: Int J Mol Sci. 2022 Dec 27;24(1):453. doi: 10.3390/ijms24010453 (PMC9820630; doi:10.3390/ijms24010453)
Supplement: Supplementary file 1 [file ijms-24-00453-s001.zip › ijms-2000310-supplementary.pdf]

Supplementary material

Figure S1. Summary of lipid metabolism.

List S1. Main abbreviations used for lipid species.

Table S1. List of detected lipids in leaves of both *ndufs4* and Col0 (Arabidopsis) with loadings of multivariate statistics (p_{corr}) and *P*-value from univariate statistics.

Table S2. List of detected lipids in leaves of both *ndufs8* and Col0 (Arabidopsis) with loadings of multivariate statistics (p_{corr}) and *P*-value from univariate statistics.

Table S3. List of detected lipids in leaves of both *CMSII* and WT (tobacco) with loadings of multivariate statistics (p_{corr}) and *P*-value from univariate statistics.

Table S4. List of detected lipids in seeds of both *ndufs4* and Col0 (Arabidopsis) with loadings of multivariate statistics (p_{corr}) and *P*-value from univariate statistics.

Table S5. List of detected lipids in seeds of both *ndufs8* and Col0 (Arabidopsis) with loadings of multivariate statistics (p_{corr}) and *P*-value from univariate statistics.

Table S6. List of detected lipids in seeds of both *CMSII* and WT (tobacco) with loadings of multivariate statistics (p_{corr}) and *P*-value from univariate statistics.

Table S7. List of detected lipids in pollen of both *CMSII* and WT (tobacco) with loadings of multivariate statistics (p_{corr}) and *P*-value from univariate statistics.

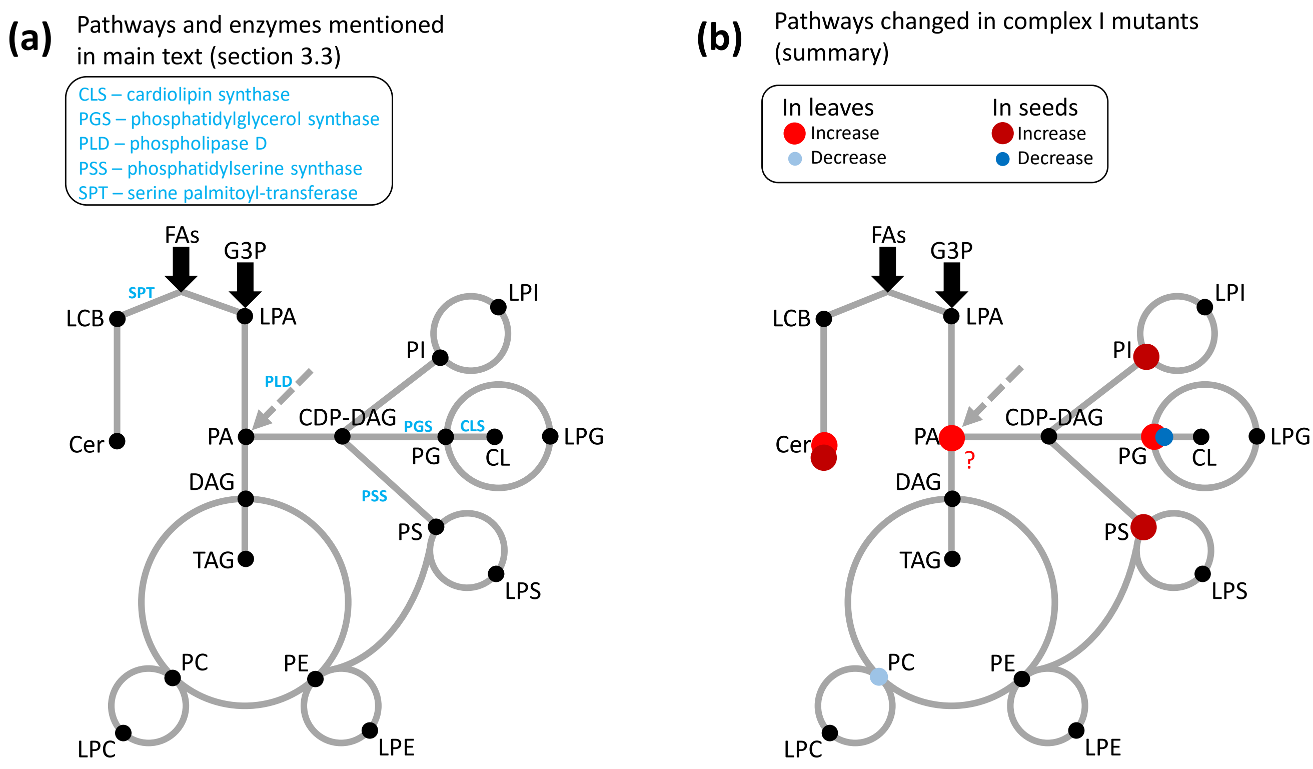

Figure S1. **Summary of lipid biosynthetic pathways and observed changes in complex I mutants**. (a) pathways with enzymes mentioned in text (in blue). (b) overview of changes observed in mutants with increases (red) and decreases (blue). For simplicity, the biosynthesis of galactolipids (chloroplast) is not shown here. Cer, ceramides; CL, cardiolipins; DAG, diacylglycerol; FAs, fatty acids; G3P, glycerol 3-phosphate; LCB, long chain base; PA, phosphatidic acid; PC, phosphatidyl choline; PE, phosphatidyl ethanolamine; PG, phosphatidyl glycerol; PI, phosphatidyl inositol; PS, phosphatidyl serine; TAG, triglycerides (triacylglycerol). Upper case 'L' stands for "lyso". Grey circles indicate the possibility of cycling between species shown. Phospholipid cleavage and recycling can generate PA, and it is shown with a dashed grey arrow. The link between PE and PS represents the interconversion between PE and PS. In (b), the question mark symbolises uncertainty about the increase in PA (see main text). Changes in pollen (which are limited to data collected in tobacco) are not shown here.

List S1. Main abbreviations used for lipid species in the paper.

Abbreviation	Meaning
LPC	Lyso phosphatidyl-choline
PC	Phosphatidyl-choline
LPG	Lyso phosphatidyl-glycerol
PG	Phosphatidyl-glycerol
LPE	Lyso phosphatidyl-ethanolamine
PE	Phosphatidyl-ethanolamine
dMePE	Dimethyl phosphatidyl-ethanolamine
LPI	Lyso phosphatidyl-inositol
PI	Phosphatidyl-inositol
PL	Phospholipids
PS	Phosphatidyl-serine
DG	Diacylglycerol (diglycerides)
TG	Triacylglycerol (triglycerides)
PA	Phosphatidic acid
PMe	Phosphatidyl-methanol
PEt	Phosphatidyl-ethanol
CL	Cardiolipin
Cer	Ceramide
MGDG	Monogalactosyl-diacylglycerol
DGDG	Digalactosyl-diacylglycerol
StE	Stigmasterol ester
PX(n:i, m:j)	Phosphatidyl-X esterified with C_n and C_m fatty acid chains, with i and j unsaturations, respectively. Example, PC(18:3, 18:3): phosphatidyl-choline esterified with two C_{18} chains carrying 3 unsaturations (linolenic type fatty acid).

Table S1 : List of p_{corr} from OPLS-DA and *P*-value from Welsch T-test of *Arabidopsis thaliana* leaf samples *ndufs4* vs col0

ID	cat	family	pcorr	pvalue
StE(32:3)	Sterols	StE	-9.99E-01	1.32E-10
SQDG(16:0_18:2)	GL	SQDG	-9.72E-01	4.85E-06
MGDG(36:7)	GL	MGDG	-9.71E-01	5.30E-06
PG(16:0_16:1)	PL	PG	-9.61E-01	1.16E-05
PG(32:3)	PL	PG	-9.57E-01	2.21E-05
SQDG(34:5)	GL	SQDG	-9.50E-01	3.05E-05
PC(16:0_22:5)	PL	PC	-9.33E-01	8.96E-05
DGDG(16:0_18:2)	GL	DGDG	-9.17E-01	1.76E-04
MGDG(16:3_18:1)	GL	MGDG	-9.08E-01	3.84E-04
DG(18:3_20:2)	DG	DG	-8.97E-01	6.04E-04
MGDG(16:0_16:3)	GL	MGDG	-8.95E-01	6.35E-04
SiE(18:2)	Sterols	SiE	-8.94E-01	4.80E-04
SiE(18:3)	Sterols	SiE	-8.86E-01	7.40E-04
StE(18:2)	Sterols	StE	-8.86E-01	7.41E-04
CL(72:12)	PL	CL	-8.83E-01	1.21E-03
PG(16:0_18:1)	PL	PG	-8.74E-01	1.15E-03
MGDG(16:0_18:3)	GL	MGDG	-8.74E-01	1.21E-03
DGDG(16:3_18:3)	GL	DGDG	-8.72E-01	8.24E-04
DGDG(16:0_18:1)	GL	DGDG	-8.60E-01	1.41E-03
SQDG(18:2_18:3)	GL	SQDG	-8.52E-01	1.88E-03
DGDG(16:0_16:0)	GL	DGDG	-8.51E-01	2.04E-03
CL(18:3_18:2_14:0_22:6)	PL	CL	-8.38E-01	3.33E-03
PE(24:1_18:3)	PL	PE	-8.30E-01	3.99E-03
MGDG(16:1_18:1)	GL	MGDG	-8.18E-01	4.29E-03
MGDG(16:1_16:3)	GL	MGDG	-8.18E-01	4.80E-03
PI(16:1_18:3)	PL	PI	-8.03E-01	7.35E-03
SQDG(16:1_18:3)	GL	SQDG	-7.89E-01	7.30E-03
DGDG(18:3_20:3)	GL	DGDG	-7.71E-01	6.68E-03
TG(18:0_16:0_18:3)	TG	TG	-7.65E-01	1.39E-02
PG(16:1_18:3)	PL	PG	-7.49E-01	1.01E-02
MGDG(16:3_18:3)	GL	MGDG	-7.32E-01	1.40E-02
MGDG(16:2_18:3)	GL	MGDG	-7.32E-01	1.89E-02
MGDG(16:0_18:1)	GL	MGDG	-7.31E-01	1.69E-02
CerG1(d18:1+hO_25:0+O)	Cer	CerG1	-7.23E-01	2.58E-02
SQDG(18:3_18:3)	GL	SQDG	-7.08E-01	2.27E-02
MGDG(16:3_16:3)	GL	MGDG	-6.88E-01	2.61E-02
CerG1(d18:1+hO_27:0+O)	Cer	CerG1	-6.87E-01	3.35E-02
CerG1(d43:2+2O)	Cer	CerG1	-6.82E-01	3.92E-02
DGDG(16:0_18:3)	GL	DGDG	-6.71E-01	3.22E-02
Cer(d18:2_27:0+O)	Cer	Cer	-6.68E-01	3.26E-02
DG(18:3_18:3)	DG	DG	-6.50E-01	5.10E-02
PI(15:0_18:3)	PL	PI	-6.12E-01	7.37E-02
PE(18:3_18:3)	PL	PE	-6.01E-01	7.88E-02
Cer(d18:2_26:0+O)	Cer	Cer	-6.00E-01	6.94E-02
PC(34:6)	PL	PC	-5.97E-01	7.70E-02
CL(18:3_18:2_16:1_20:4)	PL	CL	-5.92E-01	7.89E-02
DGDG(18:3_19:2)	GL	DGDG	-5.82E-01	8.25E-02
MGDG(18:3_20:3)	GL	MGDG	-5.65E-01	9.05E-02
PE(24:1_18:2)	PL	PE	-5.63E-01	9.99E-02
MGDG(17:3_18:3)	GL	MGDG	-5.58E-01	1.05E-01
PC(34:5)	PL	PC	-5.40E-01	1.20E-01
DG(16:0_18:3)	DG	DG	-5.34E-01	1.25E-01
PG(16:1_18:1)	PL	PG	-5.11E-01	1.26E-01
CerG1(d18:1+hO_23:0+O)	Cer	CerG1	-5.04E-01	1.70E-01
DGDG(16:0_18:0)	GL	DGDG	-4.88E-01	1.79E-01
PC(16:1_18:3)	PL	PC	-4.88E-01	1.78E-01
DG(18:3_18:2)	DG	DG	-4.84E-01	1.72E-01
DGDG(17:0_18:2)	GL	DGDG	-4.76E-01	1.81E-01
PC(38:4)	PL	PC	-4.75E-01	1.52E-01
TG(16:0_18:1_18:1)	TG	TG	-4.21E-01	2.46E-01
TG(18:1_18:1_18:2)	TG	TG	-3.98E-01	2.77E-01
PE(26:0_18:2)	PL	PE	-3.81E-01	2.83E-01
PI(18:3_18:3)	PL	PI	-3.60E-01	3.37E-01
MGDG(18:3_20:2)	GL	MGDG	-3.48E-01	3.40E-01
PG(16:0_16:0)	PL	PG	-3.47E-01	3.39E-01
PE(18:3_18:2)	PL	PE	-3.30E-01	3.70E-01
CL(70:5)	PL	CL	-3.29E-01	3.90E-01
PG(18:4_18:3)	PL	PG	-3.12E-01	3.44E-01
TG(18:1_18:1_18:1)	TG	TG	-2.51E-01	5.49E-01
PE(22:0_18:3)	PL	PE	-2.36E-01	5.49E-01
DGDG(17:0_18:3)	GL	DGDG	-2.25E-01	5.45E-01
PI(16:0_18:2)	PL	PI	-2.06E-01	5.84E-01
PE(16:0_18:3)	PL	PE	-1.87E-01	6.51E-01
Cer(d18:1+hO_26:0)	Cer	Cer	-1.77E-01	6.25E-01
DGDG(18:3_20:0)	GL	DGDG	-1.63E-01	6.36E-01
DGDG(16:2_18:3)	GL	DGDG	-1.48E-01	6.97E-01
PC(16:0_18:3)	PL	PC	-9.84E-02	8.01E-01
PC(33:1)	PL	PC	-7.67E-02	8.93E-01
MGDG(18:2_18:3)	GL	MGDG	-6.80E-02	8.99E-01
TG(18:0_16:0_18:1)	TG	TG	-3.23E-02	9.30E-01
MGDG(18:1_18:3)	GL	MGDG	-1.93E-02	9.93E-01
DG(24:0_18:2)	DG	DG	6.07E-03	9.58E-01
AGlcSiE(18:3)	AGlcSiE	AGlcSiE	2.30E-02	9.40E-01
PC(33:2)	PL	PC	8.56E-02	7.64E-01
MGDG(18:3_19:2)	GL	MGDG	1.43E-01	6.63E-01
PC(33:3)	PL	PC	1.52E-01	6.21E-01
So(d22:0+pO)	Sterols	So	1.63E-01	7.22E-01
DG(15:0_18:2)	DG	DG	1.78E-01	6.09E-01
PC(18:3_18:3)	PL	PC	1.86E-01	5.82E-01
PC(18:3_18:2)	PL	PC	1.86E-01	6.30E-01
PE(20:2_18:2)	PL	PE	2.36E-01	5.10E-01
CerG1(d42:2+2O)	Cer	CerG1	2.86E-01	3.85E-01
TG(16:0_16:0_18:1)	TG	TG	3.06E-01	3.81E-01
PE(18:2_18:2)	PL	PE	3.40E-01	3.33E-01
PC(32:1)	PL	PC	3.54E-01	2.98E-01
DGDG(18:3_18:3)	GL	DGDG	3.54E-01	3.22E-01
Cer(d18:1+hO_25:0)	Cer	Cer	3.57E-01	2.99E-01
CerG1(d18:1+hO_24:0+O)	Cer	CerG1	3.65E-01	2.73E-01
PE(17:0_18:2)	PL	PE	3.83E-01	2.59E-01
DG(18:1_18:3)	DG	DG	3.89E-01	2.60E-01
WE(5:0_17:3)	WE	WE	3.90E-01	2.92E-01
DG(18:0_20:0)	DG	DG	4.19E-01	2.36E-01
DG(18:1_18:2)	DG	DG	4.37E-01	1.78E-01
PE(24:0_18:2)	PL	PE	4.72E-01	1.59E-01
DG(18:0_18:0)	DG	DG	4.86E-01	1.51E-01
DG(16:0_16:0)	DG	DG	4.97E-01	1.44E-01
PI(18:0_18:2)	PL	PI	5.01E-01	1.27E-01
DGDG(18:1_18:3)	GL	DGDG	5.02E-01	1.32E-01
MGDG(18:0_18:2)	GL	MGDG	5.04E-01	1.15E-01
CL(18:2_16:0_16:0_20:1)	PL	CL	5.07E-01	1.37E-01
Cer(d18:0+pO_26:0)	Cer	Cer	5.08E-01	1.36E-01
PE(22:0_18:2)	PL	PE	5.11E-01	1.25E-01
So(d20:0+pO)	Sterols	So	5.46E-01	1.21E-01
PE(15:0_18:2)	PL	PE	5.50E-01	9.27E-02
PI(18:3_18:2)	PL	PI	5.53E-01	9.78E-02
PE(16:0_18:2)	PL	PE	5.78E-01	7.84E-02
TG(18:0_16:0_18:0)	TG	TG	5.96E-01	6.86E-02
PE(18:0_18:3)	PL	PE	6.02E-01	5.30E-02
DG(16:0_18:1)	DG	DG	6.04E-01	6.13E-02
DG(16:0_18:2)	DG	DG	6.09E-01	6.53E-02
DG(18:0_16:0)	DG	DG	6.16E-01	5.83E-02
StE(30:3)	Sterols	StE	6.30E-01	3.61E-02
CerG1(d18:1+hO_22:0+O)	Cer	CerG1	6.40E-01	4.03E-02
PC(16:0_18:1)	PL	PC	6.51E-01	4.03E-02
PI(16:0_18:1)	PL	PI	6.72E-01	2.94E-02
AGlcSiE(18:2)	AGlcSiE	AGlcSiE	6.89E-01	2.90E-02
DG(18:0_18:2)	DG	DG	6.91E-01	2.35E-02
AGlcSiE(18:1)	AGlcSiE	AGlcSiE	6.97E-01	2.12E-02
DG(17:0_18:2)	DG	DG	6.97E-01	2.13E-02
CerG1(d18:1+hO_28:0+O)	Cer	CerG1	7.06E-01	2.49E-02
TG(18:0_18:0_18:0)	TG	TG	7.09E-01	2.19E-02
PG(18:3_18:3)	PL	PG	7.27E-01	1.69E-02
PE(18:0_18:2)	PL	PE	7.32E-01	1.42E-02
TG(18:0_16:0_16:0)	TG	TG	7.34E-01	1.49E-02
PG(16:1_18:2)	PL	PG	7.51E-01	1.18E-02
AGlcSiE(18:0)	AGlcSiE	AGlcSiE	7.55E-01	9.42E-03
MGDG(18:3_18:3)	GL	MGDG	7.65E-01	8.34E-03
DGDG(18:0_18:3)	GL	DGDG	7.93E-01	4.97E-03
PG(18:3_18:2)	PL	PG	8.01E-01	6.79E-03
DG(18:4_18:3)	DG	DG	8.03E-01	3.96E-03

MGDG(18:0_18:3)	GL	MGDG	8.08E-01	3.37E-03
Cer(d18:1+hO_24:0)	Cer	Cer	8.18E-01	3.36E-03
Cer(d18:2_24:0+O)	Cer	Cer	8.27E-01	2.34E-03
PC(18:0_18:2)	PL	PC	8.36E-01	2.08E-03
PS(39:1)	PL	PS	8.42E-01	1.82E-03
TG(16:0_14:0_16:0)	TG	TG	8.71E-01	1.11E-03
AGlcSiE(16:0)	AGlcSiE	AGlcSiE	8.73E-01	7.11E-04
TG(16:0_16:0_16:0)	TG	TG	8.84E-01	6.64E-04
DGDG(18:0_18:1)	GL	DGDG	9.17E-01	1.16E-04
PC(17:1_17:1)	PL	PC	9.25E-01	1.65E-04
MGDG(18:3_20:1)	GL	MGDG	9.26E-01	8.50E-05
PE(16:0_18:1)	PL	PE	9.27E-01	9.55E-05
PC(38:3)	PL	PC	9.31E-01	8.95E-05
PC(18:1_18:3)	PL	PC	9.31E-01	1.31E-04

Table S2 : List of p_{corr} from OPLS-DA and *P*-value from Welsch T-test of *Arabidopsis thaliana* leaf samples *ndufs8* vs Col0

ID	cat	family	pcorr	pvalue
StE(32:3)	Sterols	StE	-9.98E-01	2.50E-11
SQDG(16:0_18:2)	GL	SQDG	-9.13E-01	2.65E-04
MGDG(36:7)	GL	MGDG	-9.14E-01	2.52E-04
PG(16:0_16:1)	PL	PG	-8.45E-01	2.01E-03
PG(32:3)	PL	PG	6.61E-01	3.38E-02
SQDG(34:5)	GL	SQDG	-5.71E-01	8.79E-02
PC(16:0_22:5)	PL	PC	-2.77E-01	4.10E-01
DGDG(16:0_18:2)	GL	DGDG	-9.18E-01	1.78E-04
MGDG(16:3_18:1)	GL	MGDG	-9.37E-01	6.14E-05
DG(18:3_20:2)	DG	DG	2.24E-01	5.49E-01
MGDG(16:0_16:3)	GL	MGDG	-2.69E-01	4.70E-01
SiE(18:2)	Sterols	SiE	-9.31E-01	7.41E-05
SiE(18:3)	Sterols	SiE	-9.49E-01	2.14E-05
StE(18:2)	Sterols	StE	-9.49E-01	2.13E-05
CL(72:12)	PL	CL	-4.98E-01	1.42E-01
PG(16:0_18:1)	PL	PG	-8.87E-01	6.16E-04
MGDG(16:0_18:3)	GL	MGDG	-9.17E-01	1.84E-04
DGDG(16:3_18:3)	GL	DGDG	-2.61E-01	4.58E-01
DGDG(16:0_18:1)	GL	DGDG	-9.39E-01	4.90E-05
SQDG(18:2_18:3)	GL	SQDG	-8.36E-01	2.93E-03
DGDG(16:0_16:0)	GL	DGDG	-9.66E-01	4.82E-06
CL(18:3_18:2_14:0_22:6)	PL	CL	-3.52E-01	3.16E-01
PE(24:1_18:3)	PL	PE	2.38E-01	5.18E-01
MGDG(16:1_18:1)	GL	MGDG	-8.91E-01	4.98E-04
MGDG(16:1_16:3)	GL	MGDG	-8.20E-01	3.39E-03
PI(16:1_18:3)	PL	PI	5.77E-01	8.05E-02
SQDG(16:1_18:3)	GL	SQDG	-3.32E-01	3.53E-01
DGDG(18:3_20:3)	GL	DGDG	3.82E-02	9.17E-01
TG(18:0_16:0_18:3)	TG	TG	-3.13E-01	3.87E-01
PG(16:1_18:3)	PL	PG	-7.54E-02	8.33E-01
MGDG(16:3_18:3)	GL	MGDG	-6.98E-01	2.42E-02
MGDG(16:2_18:3)	GL	MGDG	-8.85E-01	6.97E-04
MGDG(16:0_18:1)	GL	MGDG	-8.29E-01	2.88E-03
CerG1(d18:1+hO_25:0+O)	Cer	CerG1	-2.98E-01	4.01E-01
SQDG(18:3_18:3)	GL	SQDG	6.88E-01	2.85E-02
MGDG(16:3_16:3)	GL	MGDG	4.52E-01	1.98E-01
CerG1(d18:1+hO_27:0+O)	Cer	CerG1	-4.85E-01	1.59E-01
CerG1(d43:2+2O)	Cer	CerG1	-1.69E-01	6.48E-01
DGDG(16:0_18:3)	GL	DGDG	-9.33E-01	7.97E-05
Cer(d18:2_27:0+O)	Cer	Cer	-8.55E-02	8.00E-01
DG(18:3_18:3)	DG	DG	5.92E-01	6.82E-02
PI(15:0_18:3)	PL	PI	2.24E-01	5.31E-01
PE(18:3_18:3)	PL	PE	-1.04E-01	7.86E-01
Cer(d18:2_26:0+O)	Cer	Cer	3.46E-01	3.36E-01
PC(34:6)	PL	PC	4.92E-01	1.42E-01
CL(18:3_18:2_16:1_20:4)	PL	CL	-2.64E-01	4.58E-01
DGDG(18:3_19:2)	GL	DGDG	4.33E-01	2.05E-01
MGDG(18:3_20:3)	GL	MGDG	7.11E-02	8.61E-01
PE(24:1_18:2)	PL	PE	5.61E-01	9.48E-02
MGDG(17:3_18:3)	GL	MGDG	4.28E-01	2.18E-01
PC(34:5)	PL	PC	5.26E-01	1.18E-01
DG(16:0_18:3)	DG	DG	7.34E-01	1.42E-02
PG(16:1_18:1)	PL	PG	-3.19E-01	3.83E-01
CerG1(d18:1+hO_23:0+O)	Cer	CerG1	-7.91E-02	8.37E-01
DGDG(16:0_18:0)	GL	DGDG	-8.90E-01	5.32E-04
PC(16:1_18:3)	PL	PC	2.96E-01	4.18E-01
DG(18:3_18:2)	DG	DG	5.88E-01	7.42E-02
DGDG(17:0_18:2)	GL	DGDG	-7.86E-01	7.12E-03
PC(38:4)	PL	PC	1.09E-02	9.92E-01
TG(16:0_18:1_18:1)	TG	TG	-7.44E-01	1.43E-02
TG(18:1_18:1_18:2)	TG	TG	-2.49E-01	4.89E-01
PE(26:0_18:2)	PL	PE	-5.84E-02	8.81E-01
PI(18:3_18:3)	PL	PI	6.83E-01	2.80E-02
MGDG(18:3_20:2)	GL	MGDG	2.48E-01	5.01E-01
PG(16:0_16:0)	PL	PG	6.01E-01	5.83E-02
PE(18:3_18:2)	PL	PE	1.19E-01	7.43E-01
CL(70:5)	PL	CL	7.31E-01	1.55E-02
PG(18:4_18:3)	PL	PG	7.93E-01	6.81E-03
TG(18:1_18:1_18:1)	TG	TG	-8.23E-01	3.66E-03
PE(22:0_18:3)	PL	PE	6.93E-01	2.55E-02
DGDG(17:0_18:3)	GL	DGDG	-3.76E-01	2.81E-01
PI(16:0_18:2)	PL	PI	9.07E-02	8.00E-01
PE(16:0_18:3)	PL	PE	5.63E-01	8.70E-02
Cer(d18:1+hO_26:0)	Cer	Cer	-3.77E-02	9.06E-01
DGDG(18:3_20:0)	GL	DGDG	4.31E-01	2.10E-01
DGDG(16:2_18:3)	GL	DGDG	1.97E-01	5.95E-01
PC(16:0_18:3)	PL	PC	2.71E-01	4.35E-01
PC(33:1)	PL	PC	5.66E-01	9.36E-02
MGDG(18:2_18:3)	GL	MGDG	-1.18E-01	7.73E-01
TG(18:0_16:0_18:1)	TG	TG	-5.67E-01	8.74E-02
MGDG(18:1_18:3)	GL	MGDG	-8.90E-01	5.47E-04
DG(24:0_18:2)	DG	DG	5.51E-01	9.57E-02
AGlcSiE(18:3)	AGlcSiE	AGlcSiE	-6.54E-01	3.71E-02
PC(33:2)	PL	PC	6.30E-01	5.30E-02
MGDG(18:3_19:2)	GL	MGDG	7.96E-01	5.54E-03
PC(33:3)	PL	PC	6.06E-01	6.38E-02
So(d22:0+pO)	Sterols	So	-1.25E-01	7.53E-01
DG(15:0_18:2)	DG	DG	5.45E-01	1.05E-01
PC(18:3_18:3)	PL	PC	2.30E-01	5.06E-01
PC(18:3_18:2)	PL	PC	2.25E-01	5.33E-01
PE(20:2_18:2)	PL	PE	4.85E-01	1.62E-01
CerG1(d42:2+2O)	Cer	CerG1	5.30E-01	1.17E-01
TG(16:0_16:0_18:1)	TG	TG	-5.07E-01	1.37E-01
PE(18:2_18:2)	PL	PE	2.63E-01	4.65E-01
PC(32:1)	PL	PC	1.69E-01	6.39E-01
DGDG(18:3_18:3)	GL	DGDG	3.85E-01	2.67E-01
Cer(d18:1+hO_25:0)	Cer	Cer	7.88E-01	6.78E-03
CerG1(d18:1+hO_24:0+O)	Cer	CerG1	4.18E-01	2.30E-01
PE(17:0_18:2)	PL	PE	6.36E-01	4.70E-02
DG(18:1_18:3)	DG	DG	3.67E-01	3.08E-01
WE(5:0_17:3)	WE	WE	-3.75E-01	2.97E-01
DG(18:0_20:0)	DG	DG	-3.82E-01	2.77E-01
DG(18:1_18:2)	DG	DG	6.64E-01	3.67E-02
PE(24:0_18:2)	PL	PE	6.50E-01	4.12E-02
DG(18:0_18:0)	DG	DG	-1.49E-01	6.88E-01
DG(16:0_16:0)	DG	DG	-1.47E-01	6.93E-01
PI(18:0_18:2)	PL	PI	2.14E-01	5.58E-01
DGDG(18:1_18:3)	GL	DGDG	-9.00E-01	3.19E-04
MGDG(18:0_18:2)	GL	MGDG	-6.88E-01	2.72E-02
CL(18:2_16:0_16:0_20:1)	PL	CL	6.93E-01	2.71E-02
Cer(d18:0+pO_26:0)	Cer	Cer	-5.24E-01	1.16E-01
PE(22:0_18:2)	PL	PE	6.58E-01	3.85E-02
So(d20:0+pO)	Sterols	So	6.91E-02	8.28E-01
PE(15:0_18:2)	PL	PE	6.31E-01	5.07E-02
PI(18:3_18:2)	PL	PI	5.21E-01	1.21E-01
PE(16:0_18:2)	PL	PE	5.95E-01	7.06E-02
TG(18:0_16:0_18:0)	TG	TG	-6.37E-01	5.03E-02
PE(18:0_18:3)	PL	PE	-6.08E-02	8.61E-01
DG(16:0_18:1)	DG	DG	3.18E-01	3.77E-01
DG(16:0_18:2)	DG	DG	6.40E-01	4.39E-02
DG(18:0_16:0)	DG	DG	-2.89E-01	4.25E-01
StE(30:3)	Sterols	StE	4.83E-01	1.54E-01
CerG1(d18:1+hO_22:0+O)	Cer	CerG1	6.14E-01	5.81E-02
PC(16:0_18:1)	PL	PC	-8.36E-02	8.00E-01
PI(16:0_18:1)	PL	PI	1.97E-01	5.96E-01
AGlcSiE(18:2)	AGlcSiE	AGlcSiE	4.84E-02	9.10E-01
DG(18:0_18:2)	DG	DG	4.32E-01	2.17E-01
AGlcSiE(18:1)	AGlcSiE	AGlcSiE	-2.36E-01	4.99E-01
DG(17:0_18:2)	DG	DG	7.13E-01	1.98E-02
CerG1(d18:1+hO_28:0+O)	Cer	CerG1	3.50E-01	3.19E-01
TG(18:0_18:0_18:0)	TG	TG	-7.11E-01	2.32E-02
PG(18:3_18:3)	PL	PG	9.69E-01	4.46E-06
PE(18:0_18:2)	PL	PE	5.37E-01	1.12E-01
TG(18:0_16:0_16:0)	TG	TG	-6.58E-01	4.18E-02

PG(16:1_18:2)	PL	PG	9.18E-01	1.62E-04
AGlcSiE(18:0)	AGlcSiE	AGlcSiE	6.34E-01	5.24E-02
MGDG(18:3_18:3)	GL	MGDG	8.70E-01	1.07E-03
DGDG(18:0_18:3)	GL	DGDG	1.27E-01	7.27E-01
PG(18:3_18:2)	PL	PG	8.54E-01	1.74E-03
DG(18:4_18:3)	DG	DG	4.26E-01	2.20E-01
MGDG(18:0_18:3)	GL	MGDG	-4.79E-01	1.58E-01
Cer(d18:1+hO_24:0)	Cer	Cer	8.06E-01	5.31E-03
Cer(d18:2_24:0+O)	Cer	Cer	9.11E-01	2.67E-04
PC(18:0_18:2)	PL	PC	6.25E-02	8.84E-01
PS(39:1)	PL	PS	-8.01E-02	8.06E-01
TG(16:0_14:0_16:0)	TG	TG	-3.33E-01	3.61E-01
AGlcSiE(16:0)	AGlcSiE	AGlcSiE	8.34E-01	2.99E-03
TG(16:0_16:0_16:0)	TG	TG	-3.75E-01	3.00E-01
DGDG(18:0_18:1)	GL	DGDG	5.76E-02	8.89E-01
PC(17:1_17:1)	PL	PC	7.44E-01	1.46E-02
MGDG(18:3_20:1)	GL	MGDG	9.17E-01	1.73E-04
PE(16:0_18:1)	PL	PE	5.28E-01	1.24E-01
PC(38:3)	PL	PC	5.60E-01	1.01E-01
PC(18:1_18:3)	PL	PC	-8.03E-02	8.01E-01

Table S3 : List of p_{corr} from OPLS-DA and *P*-value from Welsch T-test of *Nicotiana sylvestris* leaf samples CMSII vs WT.

ID	cat	family	pvalue	pcorr
PA.18.2_18.2.N	PL	PA	1.39E-09	-9.44E-01
CerG1.d18.2_28.0_O.	Cer	CerG1	3.79E-09	-9.41E-01
PA.18.3_18.2.	PL	PA	4.16E-09	-9.34E-01
MGDG.18.2_18.3.	GL	MGDG	2.10E-08	-9.16E-01
Cer.d46.1_hO_O.	Cer	Cer	1.44E-07	-9.11E-01
CerG1.d18.2_29.0_O.	Cer	CerG1	9.78E-08	-9.06E-01
Cer.d18.1_hO_27.0_O.	Cer	Cer	1.59E-07	-9.05E-01
MGDG.18.1_18.3.	GL	MGDG	4.41E-08	-9.04E-01
CerG1.d18.2_26.0_O.	Cer	CerG1	2.07E-07	-9.02E-01
CerG1.d18.2_24.0_O.	Cer	CerG1	2.42E-07	-8.98E-01
Cer.d18.1_hO_23.0_O.	Cer	Cer	4.13E-07	-8.92E-01
Cer.d18.0_pO_22.0_O.	Cer	Cer	9.95E-07	-8.90E-01
Cer.d18.1_hO_24.0_O.	Cer	Cer	1.31E-06	-8.78E-01
PEt.32.4.	PL	PEt	2.02E-06	-8.76E-01
CerG1.d18.2_25.0_O.	Cer	CerG1	1.25E-06	-8.71E-01
MGDG.16.0_18.2.	GL	MGDG	1.22E-06	-8.65E-01
CerG1.d18.2_22.0_O.	Cer	CerG1	2.91E-06	-8.62E-01
PA.16.1_18.3.P	PL	PA	5.02E-06	-8.62E-01
Cer.d18.0_pO_24.0_O.	Cer	Cer	9.10E-06	-8.54E-01
CerG1.d18.2_20.0_O.	Cer	CerG1	3.85E-06	-8.53E-01
PA.18.2_18.2.P	PL	PA	2.09E-06	-8.53E-01
Cer.d18.1_hO_26.0.	Cer	Cer	5.76E-06	-8.45E-01
PA.16.0_18.2.N	PL	PA	7.79E-06	-8.43E-01
Cer.d18.0_pO_22.0.	Cer	Cer	1.36E-05	-8.42E-01
Cer.d18.1_hO_25.0_O.	Cer	Cer	7.54E-06	-8.40E-01
PA.17.0_18.3.	PL	PA	3.29E-06	-8.37E-01
Cer.d18.0_pO_26.0.	Cer	Cer	1.61E-05	-8.36E-01
Cer.d18.0_pO_26.0_O.	Cer	Cer	2.67E-05	-8.31E-01
Cer.d17.1_hO_22.0.	Cer	Cer	9.77E-06	-8.29E-01
CerG1.d18.2_31.0_O.	Cer	CerG1	6.37E-06	-8.29E-01
DGDG.16.2_18.3.N	GL	DGDG	1.80E-05	-8.20E-01
Cer.d18.0_pO_25.0.	Cer	Cer	3.34E-05	-8.17E-01
DG.16.0_18.2.	DG	DG	2.97E-05	-8.17E-01
PC.18.2_18.2.P	PL	PC	9.88E-06	-8.16E-01
Cer.d18.1_hO_23.0.	Cer	Cer	2.86E-05	-8.14E-01
PA.18.1_18.2.	PL	PA	1.44E-05	-8.11E-01
DG.18.1_18.3.	DG	DG	1.25E-05	-8.10E-01
Cer.d18.1_hO_22.0_O.	Cer	Cer	3.54E-05	-8.10E-01
Cer.d18.1_hO_26.0_O.	Cer	Cer	4.07E-05	-8.09E-01
MGDG.16.0_18.1.	GL	MGDG	1.82E-05	-8.09E-01
MGDG.16.2_18.3._10	GL	MGDG	2.65E-05	-8.03E-01
PG.16.1_18.2.P	PL	PG	2.85E-05	-8.01E-01
PG.16.0_18.1.P	PL	PG	1.79E-05	-8.00E-01
PG.16.1_18.3.N	PL	PG	5.85E-05	-7.93E-01
PE.18.2_18.2.	PL	PE	1.51E-05	-7.90E-01
DGDG.16.0_18.1.	GL	DGDG	5.89E-05	-7.89E-01
CerG1.d18.2_23.0_O.	Cer	CerG1	3.73E-05	-7.89E-01
DGDG.16.0_18.2.	GL	DGDG	1.07E-04	-7.72E-01
PA.16.0_18.2.P	PL	PA	8.86E-05	-7.71E-01
DGDG.18.1_18.3.	GL	DGDG	7.34E-05	-7.70E-01
PA.18.3_18.2._11.1	PL	PA	5.81E-05	-7.70E-01
PE.16.1_18.3.	PL	PE	5.81E-05	-7.70E-01
DGDG.16.3_18.3.	GL	DGDG	1.08E-04	-7.67E-01
CerG1.d44.3_pO.	Cer	CerG1	2.35E-04	-7.65E-01
Cer.d17.1_hO_26.0.	Cer	Cer	1.08E-04	-7.65E-01
CerG1.d42.0_O.	Cer	CerG1	2.76E-04	-7.60E-01
PA.16.0_18.1.N	PL	PA	1.93E-04	-7.53E-01
Cer.d17.1_hO_23.0.	Cer	Cer	1.60E-04	-7.51E-01
PG.16.0_18.1.N	PL	PG	1.53E-04	-7.48E-01
PA.18.0_18.2.	PL	PA	2.04E-04	-7.47E-01
DGDG.18.2_18.3.	GL	DGDG	1.52E-04	-7.46E-01
PE.37.1.	PL	PE	1.41E-04	-7.46E-01
DGDG.16.2_18.3.P	GL	DGDG	2.36E-04	-7.43E-01
PC.38.1.	PL	PC	1.38E-04	-7.42E-01
PI.18.3_18.2.	PL	PI	1.65E-04	-7.39E-01
DG.18.0_18.2.	DG	DG	3.21E-04	-7.34E-01
PG.16.1_18.3.P	PL	PG	2.78E-04	-7.32E-01
SQDG.18.3_18.3.	GL	SQDG	1.97E-04	-7.32E-01
TG.16.0_18.2_18.2.	TG	TG	1.52E-04	-7.30E-01
PMe.18.2_18.2.	PL	PMe	1.91E-04	-7.24E-01
Cer.d18.0_pO_24.0.	Cer	Cer	6.32E-04	-7.24E-01
PG.16.1_18.2.N	PL	PG	4.87E-04	-7.21E-01
PE.20.0_18.2.P	PL	PE	3.50E-04	-7.20E-01
PMe.16.1_18.3.	PL	PMe	6.50E-04	-7.02E-01
PE.18.2_18.2._12	PL	PE	3.93E-04	-6.98E-01
PG.18.0_16.0.	PL	PG	8.83E-04	-6.94E-01
PMe.16.0_16.0.	PL	PMe	9.58E-04	-6.93E-01
PG.34.5.	PL	PG	1.76E-03	-6.92E-01
Cer.d18.2_24.0_O.	Cer	Cer	7.09E-04	-6.90E-01
DGDG.16.0_18.0.	GL	DGDG	1.42E-03	-6.89E-01
PA.16.0_18.1.P	PL	PA	8.94E-04	-6.89E-01
Cer.d17.1_hO_25.0.	Cer	Cer	9.19E-04	-6.87E-01
PA.18.1_18.1.	PL	PA	9.33E-04	-6.86E-01
CerG1.d18.1_hO_22.0.	Cer	CerG1	1.15E-03	-6.77E-01
PE.15.0_18.3.	PL	PE	1.23E-03	-6.75E-01
PE.24.0_18.2.	PL	PE	1.19E-03	-6.72E-01
TG.18.0_17.0_18.3.	TG	TG	8.49E-04	-6.68E-01
MGDG.16.3_18.3.	GL	MGDG	1.95E-03	-6.67E-01
DGDG.18.3_18.3.	GL	DGDG	1.46E-03	-6.67E-01
PE.18.0_16.1.	PL	PE	1.48E-03	-6.64E-01
CerG1.d18.1_hO_26.0_O.	Cer	CerG1	1.50E-03	-6.64E-01
CerG1.d18.2_18.0_O.	Cer	CerG1	2.28E-03	-6.63E-01
PE.22.0_18.2.	PL	PE	1.30E-03	-6.63E-01
DGDG.18.3_20.4.	GL	DGDG	2.80E-03	-6.61E-01
PE.20.0_18.2.N	PL	PE	1.17E-03	-6.61E-01
PC.38.4.	PL	PC	1.69E-03	-6.60E-01
PMe.22.0_18.2.	PL	PMe	1.44E-03	-6.56E-01
PMe.20.0_18.2.	PL	PMe	1.41E-03	-6.55E-01
DGDG.16.0_16.3.	GL	DGDG	2.74E-03	-6.50E-01
DGDG.18.0_18.2.	GL	DGDG	2.08E-03	-6.43E-01
CerG1.d38.0_O.	Cer	CerG1	3.26E-03	-6.43E-01
PMe.16.0_16.1.	PL	PMe	3.34E-03	-6.37E-01
PC.36.1.	PL	PC	2.03E-03	-6.36E-01
PI.16.0_18.2.P	PL	PI	3.28E-03	-6.35E-01
PE.15.0_18.1.	PL	PE	2.43E-03	-6.35E-01
PI.16.0_18.2.N	PL	PI	3.16E-03	-6.34E-01
TG.18.0_18.2_18.3.	TG	TG	2.41E-03	-6.19E-01
TG.18.1_18.1_18.2.	TG	TG	2.62E-03	-6.18E-01
PA.18.0_18.1.	PL	PA	5.52E-03	-6.18E-01
PI.16.0_20.5.	PL	PI	3.70E-03	-6.11E-01
DGDG.18.0_18.1.	GL	DGDG	4.86E-03	-6.10E-01
TG.18.1_18.2_18.3.	TG	TG	2.88E-03	-6.09E-01
CerG1.d18.1_hO_23.0_O.	Cer	CerG1	4.95E-03	-6.04E-01
PC.34.1.	PL	PC	4.03E-03	-5.99E-01
PE.16.0_18.2.P	PL	PE	6.27E-03	-5.98E-01
PA.16.0_16.1.	PL	PA	8.43E-03	-5.98E-01
PA.16.1_18.3.N	PL	PA	7.34E-03	-5.97E-01
TG.16.0_18.2_18.3.	TG	TG	3.26E-03	-5.96E-01
PG.16.0_16.0.P	PL	PG	7.07E-03	-5.94E-01
PE.18.2_23.0.P	PL	PE	6.85E-03	-5.85E-01
CerG1.d18.1_hO_25.0_O.	Cer	CerG1	5.42E-03	-5.84E-01
PA.17.0_18.2.	PL	PA	5.69E-03	-5.83E-01
PMe.16.0_18.2.	PL	PMe	5.69E-03	-5.83E-01
DGDG.16.0_16.0.N	GL	DGDG	1.11E-02	-5.78E-01
PMe.16.0_18.1.	PL	PMe	6.09E-03	-5.78E-01
DG.18.0_18.0.	DG	DG	1.27E-02	-5.73E-01
TG.58.4.	TG	TG	7.04E-03	-5.69E-01
TG.18.3_18.2_18.2.	TG	TG	5.34E-03	-5.68E-01
DG.18.0_16.0.	DG	DG	1.46E-02	-5.60E-01
PMe.18.0_18.2.	PL	PMe	7.89E-03	-5.60E-01
DGDG.16.0_16.0.P	GL	DGDG	1.48E-02	-5.57E-01
CerG1.d43.3_pO.	Cer	CerG1	1.84E-02	-5.55E-01
TG.17.0_18.2_18.2.	TG	TG	6.69E-03	-5.51E-01
TG.19.0_18.2_18.2.	TG	TG	9.58E-03	-5.50E-01
PE.16.0_18.1.	PL	PE	9.71E-03	-5.50E-01
AGlcSiE.18.1.	AGlcSiE	AGlcSiE	1.36E-02	-5.38E-01
DG.18.0_18.1.	DG	DG	1.79E-02	-5.36E-01
CerG1.d18.1_hO_22.0_O.	Cer	CerG1	1.42E-02	-5.36E-01
PE.17.1_18.0.	PL	PE	1.29E-02	-5.35E-01
DG.16.0_18.1.	DG	DG	1.74E-02	-5.33E-01

SQDG.30.0.	GL	SQDG	2.00E-02	-5.29E-01	PA.15.0_18.3.P	PL	PA	8.11E-01	-5.27E-02
DGDG.16.0_18.3.	GL	DGDG	2.26E-02	-5.27E-01	DGDG.18.0_18.3.	GL	DGDG	8.91E-01	-3.45E-02
CerG1.d18.2_16.0_O.	Cer	CerG1	2.13E-02	-5.26E-01	TG.17.0_18.2_18.3.	TG	TG	7.52E-01	-3.35E-02
SQDG.16.0_16.0.	GL	SQDG	2.26E-02	-5.24E-01	LPMe.18.2.	PL	LPMe	8.43E-01	-2.67E-02
DGDG.16.1_18.3.	GL	DGDG	1.88E-02	-5.23E-01	TG.4.0_10.1_13.0.	TG	TG	9.89E-01	-2.32E-02
PG.16.0_16.0.N	PL	PG	2.24E-02	-5.23E-01	LPC.18.2.P	PL	LPC	8.93E-01	-1.84E-02
MGDG.16.0_16.2.	GL	MGDG	3.08E-02	-5.12E-01	AGlcSiE.16.0.	AGlcSiE	AGlcSiE	9.76E-01	-1.74E-02
TG.22.0_18.2_18.3.	TG	TG	1.67E-02	-5.07E-01	LPC.20.0.	PL	LPC	8.95E-01	-1.57E-02
AGlcSiE.17.0.	AGlcSiE	AGlcSiE	2.46E-02	-5.07E-01	LPA.18.1.	PL	LPA	9.42E-01	-8.12E-03
PE.17.1_18.3.	PL	PE	2.09E-02	-4.99E-01	LPMe.17.1.	PL	LPMe	9.46E-01	-6.83E-03
DGDG.17.3_18.3.	GL	DGDG	2.32E-02	-4.98E-01	PE.17.0_18.2.P	PL	PE	9.74E-01	-5.80E-03
SQDG.16.0_18.3.	GL	SQDG	2.65E-02	-4.96E-01	LPG.18.2.P	PL	LPG	9.76E-01	-2.44E-03
PEt.30.1.	PL	PEt	4.09E-02	-4.94E-01	TG.18.4_16.1_18.4.	TG	TG	9.65E-01	1.25E-03
PE.20.2_18.2.	PL	PE	1.92E-02	-4.92E-01	PC.16.0_16.0.	PL	PC	8.99E-01	1.39E-03
PA.20.1_18.2.	PL	PA	2.44E-02	-4.83E-01	DG.15.0_18.2.	DG	DG	8.81E-01	1.13E-02
MePC.38.7.	PL	MePC	4.04E-02	-4.82E-01	MGDG.16.0_16.3.	GL	MGDG	8.24E-01	1.42E-02
CerG1.d34.3_2O.	Cer	CerG1	4.41E-02	-4.75E-01	PMe.20.0_18.3.	PL	PMe	9.91E-01	1.49E-02
PI.16.0_18.3.P	PL	PI	3.23E-02	-4.75E-01	MGDG.18.0_18.1.	GL	MGDG	9.67E-01	1.70E-02
DG.16.0_16.0.	DG	DG	5.27E-02	-4.68E-01	DGDG.17.0_18.3.	GL	DGDG	9.02E-01	1.80E-02
MGDG.18.3_20.2.	GL	MGDG	3.71E-02	-4.65E-01	DGDG.18.3_19.1.	GL	DGDG	9.42E-01	2.47E-02
PC.18.2_18.2.N	PL	PC	3.65E-02	-4.65E-01	LPC.22.0.	PL	LPC	9.90E-01	2.91E-02
SQDG.36.5.	GL	SQDG	5.07E-02	-4.56E-01	LPA.16.1.	PL	LPA	7.74E-01	5.35E-02
PMe.18.0_18.1.	PL	PMe	3.48E-02	-4.54E-01	TG.18.3_18.2_18.3.	TG	TG	9.57E-01	5.59E-02
MGDG.18.0_18.2.	GL	MGDG	5.02E-02	-4.33E-01	DGDG.18.3_20.0.	GL	DGDG	8.78E-01	5.96E-02
PG.16.0_16.1.P	PL	PG	7.61E-02	-4.20E-01	PG.33.1.	PL	PG	7.60E-01	6.22E-02
PMe.18.3_18.2.	PL	PMe	5.38E-02	-4.13E-01	PC.16.0_17.0.	PL	PC	7.25E-01	7.02E-02
PE.16.0p.	PL	PE	7.14E-02	-4.13E-01	DGDG.39.7.	GL	DGDG	6.42E-01	8.93E-02
PE.16.0_18.2.N	PL	PE	6.81E-02	-4.11E-01	MGDG.18.3_20.3.	GL	MGDG	6.22E-01	1.06E-01
AGlcSiE.15.0.	AGlcSiE	AGlcSiE	7.95E-02	-4.10E-01	LPC.18.1.P	PL	LPC	7.01E-01	1.09E-01
TG.24.0_18.2_18.3.	TG	TG	5.85E-02	-4.03E-01	PE.15.0_18.2.	PL	PE	6.63E-01	1.12E-01
CerG1.d18.2_16.0.	Cer	CerG1	8.84E-02	-3.99E-01	SQMG.18.1.	GL	SQMG	7.33E-01	1.15E-01
PG.16.1_18.1.P	PL	PG	7.62E-02	-3.97E-01	DG.19.0_18.2.	DG	DG	5.89E-01	1.21E-01
PE.18.2_23.0.N	PL	PE	8.36E-02	-3.89E-01	PC.34.5.	PL	PC	6.34E-01	1.22E-01
PG.16.1_18.1.N	PL	PG	9.42E-02	-3.81E-01	DG.25.0_18.2.	DG	DG	5.99E-01	1.23E-01
PI.16.0_18.3.N	PL	PI	9.97E-02	-3.79E-01	Cer.d18.2_16.0_O.	Cer	Cer	4.71E-01	1.40E-01
PC.16.0_18.1.	PL	PC	1.01E-01	-3.64E-01	PC.24.2_10.4.	PL	PC	4.85E-01	1.42E-01
MGDG.18.3_18.3.	GL	MGDG	1.23E-01	-3.57E-01	DGMG.16.3.	GL	DGMG	4.84E-01	1.49E-01
CerG1.d18.1_hO_24.0_O.	Cer	CerG1	1.26E-01	-3.55E-01	MGDG.17.2_18.3.	GL	MGDG	5.21E-01	1.57E-01
PE.18.0_18.2.P	PL	PE	1.15E-01	-3.43E-01	AGlcSiE.18.0.	AGlcSiE	AGlcSiE	5.02E-01	1.61E-01
DG.18.4_16.1.	DG	DG	1.65E-01	-3.36E-01	PE.35.5.	PL	PE	5.42E-01	1.61E-01
PG.16.0_16.1.N	PL	PG	1.85E-01	-3.26E-01	PA.18.3_18.3.P	PL	PA	4.87E-01	1.73E-01
MePC.38.6p.	PL	MePC	2.40E-01	-3.24E-01	PE.34.5.	PL	PE	4.87E-01	1.73E-01
AGlcSiE.16.1.	AGlcSiE	AGlcSiE	1.89E-01	-3.21E-01	LPMe.18.1.	PL	LPMe	4.57E-01	1.91E-01
TG.18.0_18.0_18.0.	TG	TG	1.91E-01	-3.17E-01	TG.15.0_18.2_18.2.	TG	TG	4.35E-01	2.11E-01
SQDG.36.3.	GL	SQDG	1.56E-01	-3.15E-01	PA.16.0_18.3._11.9	PL	PA	3.17E-01	2.17E-01
PA.20.0_18.2.	PL	PA	3.28E-01	-2.91E-01	PE.16.1_16.1.	PL	PE	3.17E-01	2.17E-01
SQMG.18.2.	GL	SQMG	2.16E-01	-2.86E-01	LPG.18.3.P	PL	LPG	2.79E-01	2.43E-01
AGlcSiE.20.0.	AGlcSiE	AGlcSiE	2.25E-01	-2.82E-01	DG.18.1_18.2.	DG	DG	2.76E-01	2.48E-01
PC.18.0_18.1.	PL	PC	2.09E-01	-2.74E-01	MGDG.16.0_18.3.	GL	MGDG	2.56E-01	2.51E-01
LPG.16.1.P	PL	LPG	3.01E-01	-2.68E-01	PE.18.3_18.2._11	PL	PE	3.82E-01	2.53E-01
PE.18.0_18.2.N	PL	PE	2.38E-01	-2.62E-01	SiE.18.3._19.6	Sterol	SiE	2.82E-01	2.64E-01
AGlcSiE.25.0.	AGlcSiE	AGlcSiE	2.79E-01	-2.54E-01	DGDG.38.7.	GL	DGDG	2.13E-01	2.70E-01
LPI.16.0.	PL	LPI	2.92E-01	-2.52E-01	MGDG.17.3_18.3.	GL	MGDG	2.31E-01	2.88E-01
AGlcSiE.23.0.	AGlcSiE	AGlcSiE	2.72E-01	-2.50E-01	PMe.16.0_18.3.	PL	PMe	2.17E-01	3.02E-01
PE.22.0_18.3.P	PL	PE	2.49E-01	-2.48E-01	PC.24.0_18.3.P	PL	PC	2.81E-01	3.07E-01
LPI.18.2.	PL	LPI	2.91E-01	-2.39E-01	LPG.16.1.N	PL	LPG	1.67E-01	3.16E-01
MGMG.16.2.	GL	MGMG	3.02E-01	-2.35E-01	PA.16.0_18.3.	PL	PA	1.49E-01	3.16E-01
PC.34.0.	PL	PC	4.46E-01	-2.00E-01	LPG.18.2.N	PL	LPG	1.75E-01	3.28E-01
LPA.18.2.	PL	LPA	3.87E-01	-1.96E-01	MGDG.18.3_19.1.	GL	MGDG	1.63E-01	3.30E-01
AGlcSiE.22.0.	AGlcSiE	AGlcSiE	4.03E-01	-1.94E-01	PA.18.3_18.3.N	PL	PA	1.41E-01	3.46E-01
DG.18.2_23.0.	DG	DG	4.78E-01	-1.78E-01	PS.20.0_18.2.P	PL	PS	1.07E-01	3.52E-01
PC.18.3_18.2.P	PL	PC	4.03E-01	-1.72E-01	TG.12.0e_11.3_11.3.	TG	TG	1.05E-01	3.59E-01
PC.32.0.	PL	PC	5.52E-01	-1.72E-01	PC.18.3_18.2.N	PL	PC	1.29E-01	3.61E-01
DG.14.0_18.3.	DG	DG	5.53E-01	-1.71E-01	DGMG.16.0.	GL	DGMG	7.83E-02	3.99E-01
PC.18.0_18.2.	PL	PC	4.49E-01	-1.68E-01	LPMe.16.0.	PL	LPMe	8.99E-02	3.99E-01
PE.18.3_18.2.	PL	PE	4.07E-01	-1.60E-01	PE.22.0_18.3.N	PL	PE	1.23E-01	4.05E-01
PC.18.0_18.3.P	PL	PC	4.96E-01	-1.51E-01	PC.40.3.	PL	PC	1.26E-01	4.14E-01
PC.16.0_18.2.	PL	PC	5.36E-01	-1.49E-01	PE.20.0_18.3.P	PL	PE	9.07E-02	4.24E-01
DGDG.37.7.	GL	DGDG	5.43E-01	-1.46E-01	DG.26.0_18.2.	DG	DG	6.65E-02	4.25E-01
TG.16.0_17.0_18.3.	TG	TG	4.97E-01	-1.45E-01	LPC.16.0.P	PL	LPC	5.46E-02	4.32E-01
MGMG.18.2.	GL	MGMG	5.18E-01	-1.37E-01	TG.16.1_18.3_18.3.	TG	TG	7.61E-02	4.34E-01
DG.17.0_18.2.	DG	DG	6.50E-01	-1.29E-01	PC.33.2.	PL	PC	6.56E-02	4.37E-01
DG.16.0_22.0.	DG	DG	7.15E-01	-9.79E-02	MGDG.16.0_16.0.	GL	MGDG	4.79E-02	4.38E-01

DG.15.0_18.3.	DG	DG	4.84E-02	4.39E-01
TG.18.3_18.3_20.2.	TG	TG	7.20E-02	4.41E-01
DG.16.0_24.0.	DG	DG	4.25E-02	4.43E-01
PMe.18.3_18.3.	PL	PMe	6.07E-02	4.45E-01
LPC.18.0.P	PL	LPC	4.61E-02	4.46E-01
MGDG.18.0_18.3.	GL	MGDG	4.38E-02	4.51E-01
TG.18.3_17.1_18.3.	TG	TG	6.61E-02	4.57E-01
LPC.18.1.N	PL	LPC	5.07E-02	4.64E-01
PMe.18.0_18.3.	PL	PMe	4.45E-02	4.66E-01
PA.15.0_18.3.N	PL	PA	3.79E-02	4.70E-01
DG.22.0_18.2.	DG	DG	4.01E-02	4.70E-01
DG.18.1_24.0.	DG	DG	3.64E-02	4.72E-01
LPG.18.0.	PL	LPG	3.49E-02	4.72E-01
TG.16.0_16.1_18.3.	TG	TG	4.06E-02	4.77E-01
PE.38.5.	PL	PE	5.30E-02	4.79E-01
DGDG.18.3_19.0.	GL	DGDG	3.08E-02	4.86E-01
DG.24.0_18.2.	DG	DG	3.26E-02	4.86E-01
DGDG.18.2_24.0.	GL	DGDG	4.82E-02	4.89E-01
TG.15.0_18.2_18.3.	TG	TG	3.48E-02	5.04E-01
LPG.16.0.	PL	LPG	2.42E-02	5.08E-01
DG.16.0_16.1.	DG	DG	1.70E-02	5.08E-01
LPC.18.2.N	PL	LPC	2.65E-02	5.14E-01
TG.16.0_18.3_18.3.	TG	TG	2.65E-02	5.14E-01
LPE.18.2.	PL	LPE	2.69E-02	5.16E-01
PE.17.0_18.2.N	PL	PE	3.56E-02	5.17E-01
DGDG.18.2_22.0.	GL	DGDG	4.02E-02	5.18E-01
PC.8.0e_16.0.	PL	PC	2.74E-02	5.27E-01
LPC.24.0.	PL	LPC	2.53E-02	5.33E-01
StE.18.2.	Sterol	StE	2.78E-02	5.39E-01
TG.17.0_18.3_18.3.	TG	TG	1.72E-02	5.43E-01
DG.16.1_18.3.	DG	DG	8.08E-03	5.57E-01
MGMG.16.1.	GL	MGMG	1.56E-02	5.60E-01
dMePE.18.3_18.2.	PL	dMePE	1.67E-02	5.60E-01
PE.20.3_18.2.	PL	PE	1.67E-02	5.60E-01
DG.18.4_18.3.	DG	DG	9.76E-03	5.65E-01
LPG.18.1.P	PL	LPG	1.13E-02	5.69E-01
DG.26.0_16.0.	DG	DG	6.45E-03	5.71E-01
DG.18.3_20.3.	DG	DG	7.69E-03	5.71E-01
PC.33.1.	PL	PC	8.68E-03	5.73E-01
MGMG.16.3.	GL	MGMG	7.13E-03	5.75E-01
LPG.18.3.N	PL	LPG	9.49E-03	5.75E-01
PC.19.0_18.2.	PL	PC	9.33E-03	5.87E-01
SQMG.18.0.	GL	SQMG	7.17E-03	5.94E-01
DG.18.3_18.3.	DG	DG	5.25E-03	5.98E-01
PE.18.3_20.2.	PL	PE	1.16E-02	5.99E-01
DG.19.1_16.0.	DG	DG	3.62E-03	6.10E-01
LPE.16.0.	PL	LPE	5.03E-03	6.14E-01
LPMe.18.3.	PL	LPMe	3.46E-03	6.15E-01
PS.20.0_18.2.N	PL	PS	2.15E-03	6.23E-01
CerG1.d43.0_pO_2O.	Cer	CerG1	3.94E-03	6.26E-01
PE.20.0_18.3.N	PL	PE	5.66E-03	6.32E-01
PC.16.0_16.1.	PL	PC	2.89E-03	6.37E-01
MGDG.16.0_22.0.	GL	MGDG	3.80E-03	6.40E-01
DG.17.1_18.3.	DG	DG	2.30E-03	6.40E-01
PE.18.0_18.3.N	PL	PE	3.98E-03	6.44E-01
SiE.18.2.	Sterol	SiE	3.33E-03	6.53E-01
SQMG.16.0.	GL	SQMG	1.98E-03	6.54E-01
StE.25.2.	Sterol	StE	1.70E-03	6.57E-01
TG.53.9.	TG	TG	1.79E-03	6.65E-01
PC.35.1.	PL	PC	2.05E-03	6.67E-01
TG.14.0_18.3_18.3.	TG	TG	1.16E-03	6.70E-01
MGDG.16.0_24.0.	GL	MGDG	1.91E-03	6.72E-01
TG.18.3_18.3_18.3.	TG	TG	1.53E-03	6.78E-01
TG.15.0_18.3_18.3.	TG	TG	1.45E-03	6.78E-01
MGDG.17.0_18.3.	GL	MGDG	8.72E-04	6.80E-01
DG.20.0_18.3.	DG	DG	8.53E-04	6.85E-01
TG.19.1_18.3_18.3.	TG	TG	1.44E-03	6.89E-01
LPA.16.0.	PL	LPA	7.02E-04	6.89E-01
LPMe.15.0.	PL	LPMe	6.94E-04	6.89E-01
LPA.18.3.	PL	LPA	7.73E-04	6.92E-01
PC.24.0_18.3.N	PL	PC	2.31E-03	7.02E-01
PC.20.1_18.2.	PL	PC	7.37E-04	7.03E-01
PC.17.0_18.2.	PL	PC	7.06E-04	7.08E-01
PE.16.0_18.3.P	PL	PE	7.91E-04	7.10E-01
TG.18.3_18.3_20.3.	TG	TG	6.20E-04	7.10E-01
MGMG.16.0.	GL	MGMG	3.49E-04	7.13E-01
PC.16.0_18.3.P	PL	PC	2.42E-04	7.28E-01
MGMG.18.3.	GL	MGMG	3.07E-04	7.29E-01
DG.30.0_18.0.	DG	DG	2.05E-04	7.32E-01
TG.18.4_18.3_18.3.	TG	TG	3.94E-04	7.34E-01
TG.18.3_13.0_20.4.	TG	TG	2.90E-04	7.37E-01
AGlcSiE.18.3.	AGlcSiE	AGlcSiE	2.25E-04	7.38E-01
LPE.18.0.	PL	LPE	2.75E-04	7.39E-01
DG.24.0_18.3.	DG	DG	2.42E-04	7.46E-01
DG.28.0_16.0.	DG	DG	6.29E-05	7.54E-01
StE.18.3.	Sterol	StE	2.27E-04	7.56E-01
MGDG.16.0_18.0.	GL	MGDG	1.63E-04	7.57E-01
LPG.18.1.N	PL	LPG	2.62E-04	7.58E-01
DGMG.18.3.	GL	DGMG	1.45E-04	7.59E-01
DG.22.0_18.3.	DG	DG	1.58E-04	7.62E-01
PS.20.0_18.3.P	PL	PS	7.22E-05	7.64E-01
DG.19.1_18.3.	DG	DG	6.72E-05	7.66E-01
DG.30.0_16.0.	DG	DG	4.87E-05	7.67E-01
LPA.18.0.	PL	LPA	5.10E-05	7.71E-01
MGMG.18.0.	GL	MGMG	5.31E-05	7.74E-01
PC.32.3.	PL	PC	9.59E-05	7.77E-01
LPC.16.0.N	PL	LPC	7.71E-05	7.77E-01
DG.18.3_23.0.	DG	DG	6.07E-05	7.79E-01
DG.16.0_18.3.	DG	DG	3.60E-05	7.80E-01
LPC.18.0.N	PL	LPC	7.29E-05	7.80E-01
LPS.18.2.	PL	LPS	4.15E-05	7.83E-01
ChE.18.3.	Sterol	ChE	4.33E-05	7.97E-01
PE.16.0_18.3.N	PL	PE	4.56E-05	7.99E-01
ChE.18.2.	Sterol	ChE	5.33E-05	8.00E-01
LPC.17.0.	PL	LPC	2.39E-05	8.10E-01
DG.17.0_18.3.	DG	DG	1.19E-05	8.13E-01
PS.20.0_18.3.N	PL	PS	8.58E-06	8.14E-01
PE.18.3_18.3._8	PL	PE	2.46E-05	8.15E-01
PC.33.3.	PL	PC	1.56E-05	8.23E-01
PC.16.0_18.3.N	PL	PC	7.17E-06	8.26E-01
PE.18.0_18.3.P	PL	PE	8.85E-06	8.39E-01
PE.18.3_18.3.	PL	PE	1.39E-05	8.40E-01
LPI.18.3.	PL	LPI	8.18E-06	8.40E-01
DG.19.0_18.3.	DG	DG	1.51E-06	8.48E-01
PC.18.0_18.3.N	PL	PC	2.54E-06	8.50E-01
PC.19.1_16.0.	PL	PC	3.49E-06	8.60E-01
SQMG.18.3.	GL	SQMG	1.60E-06	8.62E-01
LPS.18.3.	PL	LPS	8.87E-07	8.66E-01
PS.16.0_18.3.	PL	PS	3.68E-07	8.79E-01
PE.17.0_18.3.	PL	PE	1.24E-06	8.79E-01
LPE.18.3.	PL	LPE	7.57E-07	8.81E-01
CerG1.d18.1_hO_24.0.	Cer	CerG1	8.82E-07	8.83E-01
PC.18.3_18.3.N	PL	PC	2.57E-07	8.87E-01
PS.18.0_18.3.	PL	PS	1.16E-07	8.88E-01
MGDG.18.3_24.0.	GL	MGDG	7.81E-07	8.89E-01
PC.18.3_18.3.P	PL	PC	1.91E-07	8.92E-01
LPC.18.3.N	PL	LPC	8.24E-08	9.05E-01
PC.19.0_18.3.	PL	PC	4.44E-09	9.28E-01
LPC.18.3.P	PL	LPC	1.53E-09	9.35E-01
PC.17.0_18.3.	PL	PC	8.40E-09	9.38E-01
PC.15.0_18.3.	PL	PC	3.80E-09	9.41E-01

Table S4 : List of p_{corr} from OPLS-DA and *P*-value from Welsch T-test of *Arabidopsis thaliana* seed samples *ndufs4* vs Col0

ID	family	cat	pcorr	pvalue
DGMG(18:2)	DGMG	GL	-9.97E-01	1.03E-09
DGDG(18:3_20:3)	DGDG	GL	-9.96E-01	1.57E-09
LPC(20:2)	LPC	PL	-9.95E-01	4.16E-10
DGDG(18:3_20:2)	DGDG	GL	-9.93E-01	3.91E-08
DGDG(18:2_20:1)	DGDG	GL	-9.92E-01	2.04E-08
LPC(20:3)	LPC	PL	-9.91E-01	4.97E-09
LPE(20:1)	LPE	PL	-9.91E-01	2.34E-09
LPC(20:1)	LPC	PL	-9.88E-01	2.61E-08
PG(20:1_18:2)	PG	PL	-9.88E-01	6.26E-08
DGMG(18:3)	DGMG	GL	-9.87E-01	1.39E-07
LPC(18:2)	LPC	PL	-9.87E-01	4.22E-08
LPE(18:2)	LPE	PL	-9.87E-01	1.42E-08
DGDG(16:0_18:3)	DGDG	GL	-9.86E-01	2.56E-07
PG(16:0_20:1)	PG	PL	-9.86E-01	1.93E-07
LPE(16:1)	LPE	PL	-9.84E-01	8.05E-08
LPE(24:1)	LPE	PL	-9.84E-01	2.38E-08
PE(16:0_16:0)	PE	PL	-9.84E-01	4.51E-08
DG(20:1_20:1)	DG	DG	-9.83E-01	2.42E-07
WE(19:0_17:4)	WE	WE	-9.81E-01	2.96E-07
DG(18:4_16:1)	DG	DG	-9.80E-01	5.54E-07
DGDG(18:1_18:3)	DGDG	GL	-9.80E-01	8.30E-07
LPE(22:0)	LPE	PL	-9.80E-01	6.17E-07
LPC(14:0)	LPC	PL	-9.78E-01	7.46E-07
dMePE(32:2)	dMePE	PL	-9.76E-01	4.26E-07
LPE(16:0)	LPE	PL	-9.76E-01	3.95E-07
SQDG(16:0_18:3)	SQDG	GL	-9.72E-01	1.74E-06
dMePE(18:1_18:2)	dMePE	PL	-9.71E-01	1.60E-06
LPE(18:1)	LPE	PL	-9.71E-01	6.43E-07
PE(20:1_18:2)	PE	PL	-9.71E-01	1.61E-06
PG(16:0_16:0)	PG	PL	-9.71E-01	2.72E-06
So(d18:1+hO)	So	So	-9.70E-01	3.64E-06
DG(14:0_18:3)	DG	DG	-9.69E-01	3.10E-06
DG(18:2_23:0)	DG	DG	-9.68E-01	3.01E-06
Cer(d38:3)	Cer	Cer	-9.67E-01	3.81E-06
DGDG(18:0_18:2)	DGDG	GL	-9.66E-01	6.23E-06
LPG(18:2)	LPG	PL	-9.65E-01	4.88E-06
DG(15:0_18:1)	DG	DG	-9.64E-01	3.04E-06
LPC(16:0)	LPC	PL	-9.63E-01	3.99E-06
DG(17:1_18:2)	DG	DG	-9.61E-01	2.95E-06
DG(22:1_18:2)	DG	DG	-9.61E-01	5.88E-06
PG(16:0_16:1)	PG	PL	-9.60E-01	5.54E-06
PI(20:2_18:2)	PI	PL	-9.60E-01	2.38E-06
Cer(d18:2_24:0+O)	Cer	Cer	-9.59E-01	7.48E-06
DG(15:0_18:3)	DG	DG	-9.57E-01	8.17E-06
MGDG(18:1_18:2)	MGDG	GL	-9.57E-01	1.05E-05
DG(20:1_18:1)	DG	DG	-9.56E-01	7.83E-06
LPG(16:0)	LPG	PL	-9.55E-01	9.37E-06
DG(20:1_18:2)	DG	DG	-9.53E-01	1.01E-05
DGDG(16:0_18:2)	DGDG	GL	-9.50E-01	2.61E-05
DG(16:1_18:3)	DG	DG	-9.49E-01	1.65E-05
StE(24:7)	StE	Sterols	-9.49E-01	1.18E-05
DG(16:0_16:1)	DG	DG	-9.48E-01	1.57E-05
AGlcSiE(18:2)	AGlcSiE	AGlcSiE	-9.46E-01	2.14E-05
DGDG(16:1_18:3)	DGDG	GL	-9.43E-01	3.28E-05
MGDG(16:2_18:2)	MGDG	GL	-9.38E-01	4.57E-05
DG(17:1_18:3)	DG	DG	-9.36E-01	2.48E-05
DG(18:3_20:2)	DG	DG	-9.32E-01	4.16E-05
MGDG(18:2_18:2)	MGDG	GL	-9.29E-01	7.66E-05
PI(20:1_18:2)	PI	PL	-9.27E-01	3.40E-05
DG(20:1_18:3)	DG	DG	-9.24E-01	6.64E-05
WE(3:0_18:1)	WE	WE	-9.18E-01	1.05E-04
PC(42:2)	PC	PL	-9.11E-01	8.62E-05
PE(20:1_18:3)	PE	PL	-9.11E-01	1.40E-04
LPC(18:1)	LPC	PL	-9.08E-01	1.03E-04
PC(40:3)	PC	PL	-9.07E-01	1.13E-04
LPC(16:1)	LPC	PL	-9.04E-01	1.84E-04
DG(16:1_16:1)	DG	DG	-9.02E-01	1.52E-04
PI(34:5)	PI	PL	-9.00E-01	1.39E-04
WE(3:0_20:3)	WE	WE	-9.00E-01	2.16E-04
PE(22:0_18:2)	PE	PL	-8.99E-01	1.19E-04
PC(40:4)	PC	PL	-8.96E-01	1.83E-04
DG(16:0_18:2)	DG	DG	-8.95E-01	2.62E-04
Cer(d18:0+pO_24:0)	Cer	Cer	-8.90E-01	4.64E-04
PE(17:1_16:0)	PE	PL	-8.88E-01	3.14E-04
WE(3:0_20:4)	WE	WE	-8.88E-01	3.15E-04
PG(16:1_20:2)	PG	PL	-8.86E-01	3.99E-04
PC(42:3)	PC	PL	-8.83E-01	2.85E-04
LPE(24:0)	LPE	PL	-8.82E-01	4.04E-04
DG(18:1_18:2)	DG	DG	-8.81E-01	3.60E-04
PE(22:1_18:2)	PE	PL	-8.78E-01	2.53E-04
PG(16:0_18:1)	PG	PL	-8.77E-01	4.07E-04
TG(10:0_18:2_18:3)	TG	TG	-8.77E-01	4.39E-04
DG(16:0_18:1)	DG	DG	-8.76E-01	4.01E-04
TG(43:9)	TG	TG	-8.76E-01	4.02E-04
DG(24:1_18:2)	DG	DG	-8.74E-01	5.53E-04
PA(16:1_18:3)	PA	PL	-8.74E-01	4.10E-04
TG(18:3_18:2_22:3)	TG	TG	-8.74E-01	5.53E-04
Cer(d18:2_25:0+O)	Cer	Cer	-8.73E-01	6.30E-04
PE(18:2_23:0)	PE	PL	-8.73E-01	2.78E-04
Cer(d18:1+hO_27:0)	Cer	Cer	-8.71E-01	7.51E-04
PE(24:0_18:2)	PE	PL	-8.69E-01	2.96E-04
TG(18:4_16:1_18:3)	TG	TG	-8.69E-01	6.28E-04
Cer(d18:0+pO_22:0)	Cer	Cer	-8.68E-01	8.37E-04
DG(18:2_23:1)	DG	DG	-8.67E-01	6.90E-04
CerG1(d18:1+hO_26:0+O)	CerG1	Cer	-8.66E-01	6.30E-04
Cer(d18:0+pO_26:0)	Cer	Cer	-8.65E-01	7.73E-04
Cer(d18:1+hO_26:0+O)	Cer	Cer	-8.57E-01	1.18E-03
CerG1(d18:1+hO_24:0+O)	CerG1	Cer	-8.56E-01	7.97E-04
AGlcSiE(18:1)	AGlcSiE	AGlcSiE	-8.54E-01	1.04E-03
DGDG(18:3_18:3)	DGDG	GL	-8.47E-01	1.39E-03
Cer(d18:1+hO_28:0+O)	Cer	Cer	-8.42E-01	1.48E-03
Cer(d18:2_26:0+O)	Cer	Cer	-8.42E-01	1.46E-03
PI(20:3_18:2)	PI	PL	-8.38E-01	8.20E-04
PC(27:1_11:4)	PC	PL	-8.30E-01	1.40E-03
PA(18:3_18:3)	PA	PL	-8.29E-01	1.35E-03
TG(18:3_20:3_22:3)	TG	TG	-8.29E-01	1.56E-03
PE(25:0_18:2)	PE	PL	-8.24E-01	1.03E-03
DG(18:0_18:1)	DG	DG	-8.23E-01	1.72E-03
DG(17:0_18:2)	DG	DG	-8.14E-01	1.93E-03
DG(18:1_18:3)	DG	DG	-8.14E-01	2.18E-03
MGMG(18:3)	MGMG	GL	-8.14E-01	2.68E-03
DG(18:1_18:1)	DG	DG	-8.13E-01	1.95E-03
PI(34:6)	PI	PL	-8.10E-01	1.97E-03
Cer(d18:1+hO_26:0)	Cer	Cer	-8.06E-01	3.26E-03
TG(16:0_14:0_18:2)	TG	TG	-8.02E-01	2.84E-03
PC(42:4)	PC	PL	-7.96E-01	2.56E-03
DG(16:0_16:0)	DG	DG	-7.90E-01	3.38E-03
DG(17:0_18:1)	DG	DG	-7.89E-01	2.94E-03
TG(18:4_17:1_18:3)	TG	TG	-7.81E-01	4.04E-03
DG(19:1_18:3)	DG	DG	-7.60E-01	4.95E-03
So(d18:0+pO)	So	So	-7.57E-01	7.34E-03
TG(18:3_14:1_18:3)	TG	TG	-7.44E-01	7.63E-03
PC(34:6)	PC	PL	-7.38E-01	6.42E-03
LPI(16:0)	LPI	PL	-7.35E-01	7.43E-03
TG(18:1_18:2_18:3)	TG	TG	-7.29E-01	9.04E-03
TG(12:0_18:2_18:3)	TG	TG	-7.25E-01	9.57E-03
LPI(18:2)	LPI	PL	-7.22E-01	8.67E-03
TG(16:0_12:0_18:2)	TG	TG	-7.17E-01	1.06E-02
PI(14:0_18:3)	PI	PL	-7.11E-01	1.25E-02
PC(37:3)	PC	PL	-7.08E-01	9.69E-03
PE(15:0_18:2)	PE	PL	-7.03E-01	1.45E-02
TG(18:3_17:1_18:3)	TG	TG	-7.01E-01	1.24E-02
LdMePE(16:0)	LdMePE	PL	-6.80E-01	1.48E-02
AGlcSiE(16:1)	AGlcSiE	AGlcSiE	-6.79E-01	1.49E-02
DG(16:0_14:0)	DG	DG	-6.79E-01	1.61E-02
TG(18:3_18:2_20:3)	TG	TG	-6.79E-01	1.66E-02
TG(16:1_18:3_18:3)	TG	TG	-6.70E-01	1.89E-02
TG(16:0_18:1_18:2)	TG	TG	-6.62E-01	2.02E-02
PI(15:0_18:2)	PI	PL	-6.61E-01	1.81E-02
CerG1(d18:1+hO_23:0+O)	CerG1	Cer	-6.60E-01	2.00E-02

PC(30:0)	PC	PL	-6.45E-01	2.04E-02
LPE(18:0)	LPE	PL	-6.20E-01	2.41E-02
Cer(d18:0+pO_23:0)	Cer	Cer	-6.01E-01	3.81E-02
DGDG(18:2_18:3)	DGDG	GL	-5.80E-01	3.90E-02
LPS(18:2)	LPS	PL	-5.79E-01	3.58E-02
AGlcSiE(19:1)	AGlcSiE	AGlcSiE	-5.73E-01	4.21E-02
SQDG(18:2_18:3)	SQDG	GL	-5.73E-01	3.59E-02
PI(20:0_18:2)	PI	PL	-5.63E-01	3.65E-02
DG(16:1_18:2)	DG	DG	-5.62E-01	4.94E-02
PG(15:0_18:3)	PG	PL	-5.61E-01	5.35E-02
LPC(18:3)	LPC	PL	-5.56E-01	5.07E-02
Cer(d18:1+hO_24:0+O)	Cer	Cer	-5.52E-01	5.75E-02
TG(18:3_18:2_18:3)	TG	TG	-5.49E-01	5.47E-02
PC(33:2)	PC	PL	-5.40E-01	5.86E-02
MG(20:0)	MG	MG	-5.36E-01	5.13E-02
TG(19:0_18:3_18:3)	TG	TG	-5.29E-01	5.84E-02
LPI(18:1)	LPI	PL	-5.27E-01	5.18E-02
TG(14:0_18:2_18:3)	TG	TG	-5.25E-01	6.36E-02
MG(16:0)	MG	MG	-5.20E-01	5.84E-02
DG(24:1_18:3)	DG	DG	-5.11E-01	6.87E-02
PG(16:1_18:2)	PG	PL	-5.08E-01	8.10E-02
BisMePE(16:0_18:2)	BisMePE	PL	-4.92E-01	7.53E-02
LPC(15:0)	LPC	PL	-4.72E-01	9.79E-02
PA(18:1_18:2)	PA	PL	-4.71E-01	8.07E-02
dMePE(18:3_18:2)	dMePE	PL	-4.61E-01	1.03E-01
Cer(d18:0+pO_25:0)	Cer	Cer	-4.44E-01	1.14E-01
PC(16:0_18:2)	PC	PL	-4.35E-01	1.07E-01
PC(38:6)	PC	PL	-4.33E-01	1.01E-01
AGlcSiE(16:0)	AGlcSiE	AGlcSiE	-4.28E-01	1.14E-01
PS(18:1_18:2)	PS	PL	-4.26E-01	9.17E-02
PC(35:4)	PC	PL	-4.15E-01	1.03E-01
TG(18:1_12:0_18:2)	TG	TG	-3.84E-01	1.42E-01
PC(16:0_18:1)	PC	PL	-3.72E-01	1.43E-01
LPE(18:3)	LPE	PL	-3.68E-01	1.54E-01
WE(5:0_17:3)	WE	WE	-3.45E-01	1.79E-01
So(d22:0+pO)	So	So	-3.15E-01	1.93E-01
MG(18:0)	MG	MG	-2.84E-01	2.12E-01
PI(18:2_23:0)	PI	PL	-2.74E-01	1.97E-01
Cer(d18:1+hO_25:0+O)	Cer	Cer	-2.44E-01	2.69E-01
DG(18:0_18:0)	DG	DG	-2.33E-01	2.64E-01
PA(18:1_18:1)	PA	PL	-2.33E-01	2.42E-01
So(d20:0+pO)	So	So	-2.06E-01	2.95E-01
TG(12:0_18:3_18:3)	TG	TG	-1.78E-01	3.17E-01
TG(10:0_18:3_18:3)	TG	TG	-1.71E-01	3.17E-01
TG(18:3_18:3_18:3)	TG	TG	-1.26E-01	3.76E-01
PG(18:2_18:2)	PG	PL	-1.08E-01	4.09E-01
DG(18:0_16:0)	DG	DG	-7.22E-02	4.24E-01
PG(16:1_18:1)	PG	PL	-6.57E-02	4.63E-01
PC(35:3)	PC	PL	-2.05E-02	4.65E-01
PA(20:1_18:2)	PA	PL	2.48E-03	4.79E-01
dMePE(16:0_18:3)	dMePE	PL	1.06E-02	4.69E-01
PE(18:1_18:2)	PE	PL	1.58E-02	4.63E-01
PG(15:0_18:2)	PG	PL	2.85E-02	4.42E-01
PE(17:0_18:2)	PE	PL	3.43E-02	4.39E-01
MGDG(16:3_18:3)	MGDG	GL	3.82E-02	4.37E-01
PC(34:4)	PC	PL	4.25E-02	4.70E-01
DG(20:0_22:0)	DG	DG	4.96E-02	4.26E-01
PA(18:2_18:2)	PA	PL	5.24E-02	4.48E-01
PC(16:0_20:1)	PC	PL	6.52E-02	4.50E-01
Cer(d18:0_16:0)	Cer	Cer	9.06E-02	3.72E-01
PC(17:1_13:0)	PC	PL	1.36E-01	3.79E-01
AGlcSiE(17:1)	AGlcSiE	AGlcSiE	1.38E-01	3.56E-01
TG(15:0_18:2_18:3)	TG	TG	1.76E-01	3.00E-01
AGlcSiE(15:0)	AGlcSiE	AGlcSiE	1.96E-01	2.83E-01
PI(18:0_18:3)	PI	PL	2.35E-01	2.93E-01
DG(18:3_18:3)	DG	DG	2.93E-01	1.98E-01
PS(20:0_18:2)	PS	PL	3.14E-01	2.09E-01
PC(34:3)	PC	PL	3.16E-01	1.88E-01
PC(35:2)	PC	PL	3.23E-01	1.86E-01
PS(16:0_18:2)	PS	PL	3.53E-01	1.83E-01
TG(16:0_18:2_18:3)	TG	TG	3.86E-01	1.27E-01
TG(17:0_18:3_18:3)	TG	TG	4.00E-01	1.23E-01
TG(20:1_18:3_18:3)	TG	TG	4.35E-01	9.89E-02

PS(22:0_18:3)	PS	PL	4.49E-01	1.13E-01
PC(18:3_18:3)	PC	PL	4.72E-01	8.50E-02
PI(16:1_18:3)	PI	PL	4.79E-01	7.34E-02
PS(18:3_18:2)	PS	PL	4.95E-01	8.43E-02
PG(18:3_18:3)	PG	PL	5.00E-01	5.87E-02
TG(4:0_16:0_18:3)	TG	TG	5.28E-01	6.26E-02
PS(22:0_18:2)	PS	PL	5.30E-01	6.84E-02
LPC(18:0)	LPC	PL	5.34E-01	5.57E-02
LPI(18:0)	LPI	PL	5.56E-01	5.37E-02
MG(24:1)	MG	MG	5.66E-01	3.46E-02
PE(18:2_18:2)	PE	PL	5.74E-01	3.45E-02
PI(16:0_18:2)	PI	PL	5.82E-01	4.61E-02
TG(18:3_18:3_20:3)	TG	TG	5.86E-01	3.60E-02
PE(16:0_16:1)	PE	PL	6.31E-01	2.07E-02
PE(16:1_18:3)	PE	PL	6.51E-01	1.69E-02
TG(18:3_18:3_22:3)	TG	TG	6.60E-01	1.84E-02
TG(19:1_18:3_18:3)	TG	TG	6.94E-01	1.29E-02
TG(15:0_18:3_18:3)	TG	TG	6.99E-01	1.09E-02
PS(24:1_18:2)	PS	PL	7.08E-01	1.47E-02
PC(37:4)	PC	PL	7.46E-01	6.99E-03
PE(16:0_18:2)	PE	PL	7.79E-01	2.91E-03
PI(15:0_18:3)	PI	PL	8.02E-01	2.85E-03
AGlcSiE(18:3)	AGlcSiE	AGlcSiE	8.18E-01	1.66E-03
PI(18:0_20:1)	PI	PL	8.23E-01	2.48E-03
PS(20:1_18:3)	PS	PL	8.28E-01	2.32E-03
PE(18:3_22:1)	PE	PL	8.29E-01	1.29E-03
TG(16:0_18:3_18:3)	TG	TG	8.39E-01	1.03E-03
PI(18:2_23:1)	PI	PL	8.50E-01	1.34E-03
PS(18:3_22:1)	PS	PL	8.52E-01	1.31E-03
PI(14:0_18:2)	PI	PL	8.59E-01	5.55E-04
TG(18:4_15:0_18:3)	TG	TG	8.62E-01	5.84E-04
PS(18:0_18:2)	PS	PL	8.72E-01	3.20E-04
So(d18:0)	So	So	8.74E-01	3.96E-04
PI(17:0_18:2)	PI	PL	8.77E-01	6.97E-04
PI(18:3_18:2)	PI	PL	8.92E-01	3.17E-04
PG(18:3_18:2)	PG	PL	9.00E-01	9.29E-05
PE(16:0_20:1)	PE	PL	9.11E-01	2.26E-04
PI(18:0_18:2)	PI	PL	9.20E-01	1.32E-04
dMePE(16:1_18:1)	dMePE	PL	9.21E-01	7.74E-05
PE(18:1_18:1)	PE	PL	9.29E-01	5.10E-05
PS(24:1_18:3)	PS	PL	9.30E-01	9.31E-05
Cer(d16:0_19:0)	Cer	Cer	9.33E-01	4.42E-05
PE(18:3_18:2)	PE	PL	9.33E-01	2.00E-05
MGMG(16:3)	MGMG	GL	9.34E-01	5.16E-05
PI(18:0_18:1)	PI	PL	9.36E-01	6.87E-05
PE(24:1_18:2)	PE	PL	9.39E-01	4.56E-05
TG(10:0_10:1_18:1)	TG	TG	9.39E-01	2.92E-05
DGDG(16:3_18:3)	DGDG	GL	9.44E-01	1.10E-05
AGlcSiE(17:0)	AGlcSiE	AGlcSiE	9.45E-01	1.72E-05
PS(16:0_18:3)	PS	PL	9.48E-01	3.03E-05
Cer(d18:1_15:0)	Cer	Cer	9.74E-01	7.32E-07
PE(18:3_18:3)	PE	PL	9.76E-01	2.74E-07
PI(18:2_18:2)	PI	PL	9.76E-01	2.36E-06
PE(16:0_18:1)	PE	PL	9.77E-01	1.06E-06
PS(18:3_18:3)	PS	PL	9.79E-01	1.15E-06
PI(17:0_18:3)	PI	PL	9.82E-01	9.32E-07
PI(18:3_18:3)	PI	PL	9.85E-01	1.10E-07
PI(16:0_18:3)	PI	PL	9.89E-01	5.09E-08
LPI(17:0)	LPI	PL	9.91E-01	4.24E-08
dMePE(16:1_18:3)	dMePE	PL	9.92E-01	6.38E-10
DGDG(17:0_18:2)	DGDG	GL	9.93E-01	7.84E-10
LPC(17:0)	LPC	PL	9.93E-01	1.90E-09

Table S5 : List of p_{corr} from OPLS-DA and *P*-value from Welsch T-test of *Arabidopsis thaliana* seed samples *ndufs8* vs col0

ID	family	cat	pcorr	pvalue
AGlcSiE(15:0)	AGlcSiE	AGlcSiE	-2.20E-01	2.67E-01
AGlcSiE(16:0)	AGlcSiE	AGlcSiE	-5.74E-01	4.06E-02
AGlcSiE(16:1)	AGlcSiE	AGlcSiE	-4.06E-01	1.22E-01
AGlcSiE(17:0)	AGlcSiE	AGlcSiE	6.86E-01	1.45E-02
AGlcSiE(17:1)	AGlcSiE	AGlcSiE	-7.69E-01	4.13E-03
AGlcSiE(18:1)	AGlcSiE	AGlcSiE	-9.38E-01	2.89E-05
AGlcSiE(18:2)	AGlcSiE	AGlcSiE	-9.47E-01	1.52E-05
AGlcSiE(18:3)	AGlcSiE	AGlcSiE	1.29E-01	3.63E-01
AGlcSiE(19:1)	AGlcSiE	AGlcSiE	-8.83E-01	3.55E-04
BisMePE(16:0_18:2)	BisMePE	PL	-9.01E-01	1.79E-04
Cer(d16:0_19:0)	Cer	Cer	8.97E-01	2.25E-04
Cer(d18:0_16:0)	Cer	Cer	-6.66E-01	2.10E-02
Cer(d18:0+pO_22:0)	Cer	Cer	-9.35E-01	5.27E-05
Cer(d18:0+pO_23:0)	Cer	Cer	-8.90E-01	3.31E-04
Cer(d18:0+pO_24:0)	Cer	Cer	-9.32E-01	5.80E-05
Cer(d18:0+pO_25:0)	Cer	Cer	-8.91E-01	3.29E-04
Cer(d18:0+pO_26:0)	Cer	Cer	-9.35E-01	4.76E-05
Cer(d18:1_15:0)	Cer	Cer	8.63E-01	4.23E-04
Cer(d18:1+hO_24:0+O)	Cer	Cer	-6.79E-01	1.70E-02
Cer(d18:1+hO_25:0+O)	Cer	Cer	-7.17E-01	1.08E-02
Cer(d18:1+hO_26:0)	Cer	Cer	-8.78E-01	4.69E-04
Cer(d18:1+hO_26:0+O)	Cer	Cer	-8.06E-01	2.47E-03
Cer(d18:1+hO_27:0)	Cer	Cer	-9.35E-01	5.23E-05
Cer(d18:1+hO_28:0+O)	Cer	Cer	-8.87E-01	3.97E-04
Cer(d18:2_24:0+O)	Cer	Cer	-9.74E-01	1.30E-06
Cer(d18:2_25:0+O)	Cer	Cer	-9.45E-01	2.41E-05
Cer(d18:2_26:0+O)	Cer	Cer	-9.05E-01	1.93E-04
Cer(d38:3)	Cer	Cer	-8.47E-01	1.10E-03
CerG1(d18:1+hO_23:0+O)	CerG1	Cer	-8.44E-01	1.04E-03
CerG1(d18:1+hO_24:0+O)	CerG1	Cer	-9.18E-01	8.82E-05
CerG1(d18:1+hO_26:0+O)	CerG1	Cer	-9.21E-01	6.60E-05
DG(14:0_18:3)	DG	DG	-9.76E-01	1.30E-06
DG(15:0_18:1)	DG	DG	-9.35E-01	3.88E-05
DG(15:0_18:3)	DG	DG	-9.72E-01	1.21E-06
DG(16:0_14:0)	DG	DG	-6.54E-01	2.35E-02
DG(16:0_16:0)	DG	DG	-8.16E-01	2.39E-03
DG(16:0_16:1)	DG	DG	-9.33E-01	3.21E-05
DG(16:0_18:1)	DG	DG	-5.43E-01	4.94E-02
DG(16:0_18:2)	DG	DG	-8.94E-01	2.27E-04
DG(16:1_16:1)	DG	DG	-3.24E-01	1.79E-01
DG(16:1_18:2)	DG	DG	2.92E-01	2.06E-01
DG(16:1_18:3)	DG	DG	4.10E-01	1.27E-01
DG(17:0_18:1)	DG	DG	4.60E-01	9.11E-02
DG(17:0_18:2)	DG	DG	7.08E-01	1.06E-02
DG(17:1_18:2)	DG	DG	1.35E-01	3.71E-01
DG(17:1_18:3)	DG	DG	-8.51E-02	4.25E-01
DG(18:0_16:0)	DG	DG	-5.37E-01	5.91E-02
DG(18:0_18:0)	DG	DG	-5.97E-01	3.64E-02
DG(18:0_18:1)	DG	DG	-4.33E-01	1.02E-01
DG(18:1_18:1)	DG	DG	5.96E-01	3.82E-02
DG(18:1_18:2)	DG	DG	9.63E-02	4.36E-01
DG(18:1_18:3)	DG	DG	-6.19E-01	2.57E-02
DG(18:2_23:0)	DG	DG	-9.62E-01	3.84E-06
DG(18:2_23:1)	DG	DG	-9.59E-01	4.54E-06
DG(18:3_18:3)	DG	DG	9.91E-01	1.80E-08
DG(18:3_20:2)	DG	DG	-9.26E-01	3.96E-05
DG(18:4_16:1)	DG	DG	-8.82E-01	3.04E-04
DG(19:1_18:3)	DG	DG	1.75E-01	3.32E-01
DG(20:0_22:0)	DG	DG	-9.40E-01	3.10E-05
DG(20:1_18:1)	DG	DG	-8.91E-01	2.25E-04
DG(20:1_18:2)	DG	DG	-9.05E-01	1.21E-04
DG(20:1_18:3)	DG	DG	-5.80E-01	3.50E-02
DG(20:1_20:1)	DG	DG	-9.60E-01	3.88E-06
DG(22:1_18:2)	DG	DG	-9.01E-01	1.63E-04
DG(24:1_18:2)	DG	DG	-7.82E-01	3.58E-03
DG(24:1_18:3)	DG	DG	6.33E-01	2.58E-02
DGDG(16:0_18:2)	DGDG	GL	-4.24E-01	1.18E-01
DGDG(16:0_18:3)	DGDG	GL	-2.85E-01	2.17E-01
DGDG(16:1_18:3)	DGDG	GL	9.20E-01	4.51E-05
DGDG(16:3_18:3)	DGDG	GL	4.14E-01	1.10E-01
DGDG(17:0_18:2)	DGDG	GL	9.94E-01	1.25E-10
DGDG(18:0_18:2)	DGDG	GL	2.96E-01	1.90E-01
DGDG(18:1_18:3)	DGDG	GL	-8.56E-01	9.75E-04
DGDG(18:2_18:3)	DGDG	GL	3.59E-01	1.48E-01
DGDG(18:2_20:1)	DGDG	GL	-7.65E-01	7.28E-03
DGDG(18:3_18:3)	DGDG	GL	8.26E-01	1.16E-03
DGDG(18:3_20:2)	DGDG	GL	3.98E-01	1.13E-01
DGDG(18:3_20:3)	DGDG	GL	-9.42E-01	2.94E-05
DGMG(18:2)	DGMG	GL	-8.56E-01	1.16E-03
DGMG(18:3)	DGMG	GL	-6.86E-01	1.69E-02
dMePE(16:0_18:3)	dMePE	PL	-7.30E-02	4.35E-01
dMePE(16:1_18:1)	dMePE	PL	9.86E-01	1.01E-07
dMePE(16:1_18:3)	dMePE	PL	9.68E-01	8.39E-07
dMePE(18:1_18:2)	dMePE	PL	-9.48E-01	1.76E-05
dMePE(18:3_18:2)	dMePE	PL	-4.36E-01	1.15E-01
dMePE(32:2)	dMePE	PL	-9.72E-01	2.22E-06
LdMePE(16:0)	LdMePE	PL	-7.65E-01	5.18E-03
LPC(14:0)	LPC	PL	-9.55E-01	1.68E-05
LPC(15:0)	LPC	PL	-8.19E-01	2.73E-03
LPC(16:0)	LPC	PL	-6.40E-01	2.75E-02
LPC(16:1)	LPC	PL	-9.04E-01	2.64E-04
LPC(17:0)	LPC	PL	9.79E-01	1.89E-07
LPC(18:0)	LPC	PL	9.72E-01	8.61E-07
LPC(18:1)	LPC	PL	7.98E-01	2.79E-03
LPC(18:2)	LPC	PL	-8.89E-01	4.45E-04
LPC(18:3)	LPC	PL	4.11E-01	1.05E-01
LPC(20:1)	LPC	PL	4.43E-01	9.98E-02
LPC(20:2)	LPC	PL	-9.77E-01	8.60E-07
LPC(20:3)	LPC	PL	-2.08E-01	2.76E-01
LPE(16:0)	LPE	PL	-5.33E-01	6.02E-02
LPE(16:1)	LPE	PL	-9.15E-01	1.61E-04
LPE(18:0)	LPE	PL	8.83E-01	3.76E-04
LPE(18:1)	LPE	PL	7.65E-01	5.39E-03
LPE(18:2)	LPE	PL	-9.25E-01	8.25E-05
LPE(18:3)	LPE	PL	8.36E-01	9.12E-04
LPE(20:1)	LPE	PL	2.31E-01	2.69E-01
LPE(22:0)	LPE	PL	-9.70E-01	3.43E-06
LPE(24:0)	LPE	PL	-9.14E-01	1.57E-04
LPE(24:1)	LPE	PL	-9.34E-01	4.31E-05
LPG(16:0)	LPG	PL	-9.85E-01	2.75E-07
LPG(18:2)	LPG	PL	-9.90E-01	8.64E-08
LPI(16:0)	LPI	PL	-8.43E-01	1.51E-03
LPI(17:0)	LPI	PL	9.93E-01	4.62E-09
LPI(18:0)	LPI	PL	9.04E-01	1.77E-04
LPI(18:1)	LPI	PL	9.72E-01	1.38E-06
LPI(18:2)	LPI	PL	-9.29E-01	7.28E-05
LPS(18:2)	LPS	PL	-6.47E-01	2.08E-02
MG(16:0)	MG	MG	-2.57E-01	2.52E-01
MG(18:0)	MG	MG	-2.22E-01	2.74E-01
MG(20:0)	MG	MG	-5.81E-01	3.99E-02
MG(24:1)	MG	MG	6.06E-01	2.34E-02
MGDG(16:2_18:2)	MGDG	GL	8.06E-01	2.08E-03
MGDG(16:3_18:3)	MGDG	GL	1.14E-01	3.65E-01
MGDG(18:1_18:2)	MGDG	GL	-7.59E-01	6.84E-03
MGDG(18:2_18:2)	MGDG	GL	-8.52E-01	1.15E-03
MGMG(16:3)	MGMG	GL	-8.42E-01	9.02E-04
MGMG(18:3)	MGMG	GL	3.01E-01	1.93E-01
PA(16:1_18:3)	PA	PL	-9.23E-01	6.22E-05
PA(18:1_18:1)	PA	PL	5.02E-02	4.59E-01
PA(18:1_18:2)	PA	PL	-7.19E-01	8.54E-03
PA(18:2_18:2)	PA	PL	-4.41E-01	9.64E-02
PA(18:3_18:3)	PA	PL	-8.45E-01	8.64E-04
PA(20:1_18:2)	PA	PL	-1.88E-02	4.65E-01
PC(16:0_18:1)	PC	PL	-4.84E-01	7.29E-02
PC(16:0_18:2)	PC	PL	-6.62E-01	1.75E-02
PC(16:0_20:1)	PC	PL	5.83E-01	4.32E-02
PC(17:1_13:0)	PC	PL	5.47E-01	5.64E-02
PC(18:3_18:3)	PC	PL	8.88E-01	3.45E-04
PC(27:1_11:4)	PC	PL	-8.72E-01	4.21E-04
PC(30:0)	PC	PL	-8.96E-01	2.02E-04

PC(33:2)	PC	PL	-9.08E-01	1.24E-04
PC(34:3)	PC	PL	-1.99E-01	2.81E-01
PC(34:4)	PC	PL	6.16E-01	3.23E-02
PC(34:6)	PC	PL	-5.80E-01	3.48E-02
PC(35:2)	PC	PL	7.37E-01	8.12E-03
PC(35:3)	PC	PL	9.16E-01	1.24E-04
PC(35:4)	PC	PL	5.93E-02	4.44E-01
PC(37:3)	PC	PL	-9.67E-02	3.80E-01
PC(37:4)	PC	PL	8.67E-01	6.40E-04
PC(38:6)	PC	PL	7.26E-01	1.02E-02
PC(40:3)	PC	PL	-9.24E-01	4.65E-05
PC(40:4)	PC	PL	-7.50E-01	6.00E-03
PC(42:2)	PC	PL	-9.39E-01	2.45E-05
PC(42:3)	PC	PL	-8.94E-01	2.11E-04
PC(42:4)	PC	PL	-9.21E-01	5.03E-05
PE(15:0_18:2)	PE	PL	-9.61E-01	9.18E-06
PE(16:0_16:0)	PE	PL	-9.82E-01	1.54E-07
PE(16:0_16:1)	PE	PL	8.34E-01	9.47E-04
PE(16:0_18:1)	PE	PL	9.64E-01	4.59E-06
PE(16:0_18:2)	PE	PL	-8.68E-01	7.91E-04
PE(16:0_20:1)	PE	PL	9.63E-01	6.01E-06
PE(16:1_18:3)	PE	PL	7.83E-01	2.92E-03
PE(17:0_18:2)	PE	PL	9.73E-01	7.04E-07
PE(17:1_16:0)	PE	PL	-1.29E-01	3.71E-01
PE(18:1_18:1)	PE	PL	9.87E-01	8.32E-08
PE(18:1_18:2)	PE	PL	-6.86E-02	4.40E-01
PE(18:2_18:2)	PE	PL	-7.23E-01	1.08E-02
PE(18:2_23:0)	PE	PL	-9.41E-01	1.92E-05
PE(18:3_18:2)	PE	PL	7.91E-01	2.52E-03
PE(18:3_18:3)	PE	PL	9.73E-01	3.87E-07
PE(18:3_22:1)	PE	PL	-8.56E-01	1.00E-03
PE(20:1_18:2)	PE	PL	-9.48E-01	1.74E-05
PE(20:1_18:3)	PE	PL	-1.36E-01	3.64E-01
PE(22:0_18:2)	PE	PL	-9.27E-01	5.48E-05
PE(22:1_18:2)	PE	PL	-9.58E-01	4.55E-06
PE(24:0_18:2)	PE	PL	-8.84E-01	3.25E-04
PE(24:1_18:2)	PE	PL	-8.58E-01	6.93E-04
PE(25:0_18:2)	PE	PL	-8.88E-01	2.81E-04
PG(15:0_18:2)	PG	PL	-8.65E-01	8.17E-04
PG(15:0_18:3)	PG	PL	-7.84E-01	5.20E-03
PG(16:0_16:0)	PG	PL	-9.92E-01	9.11E-09
PG(16:0_16:1)	PG	PL	2.22E-01	2.69E-01
PG(16:0_18:1)	PG	PL	9.71E-01	2.44E-06
PG(16:0_20:1)	PG	PL	-9.54E-01	9.49E-06
PG(16:1_18:1)	PG	PL	-8.99E-01	2.67E-04
PG(16:1_18:2)	PG	PL	-9.27E-01	8.18E-05
PG(16:1_20:2)	PG	PL	-8.88E-01	3.26E-04
PG(18:2_18:2)	PG	PL	-9.44E-01	2.83E-05
PG(18:3_18:2)	PG	PL	-8.79E-01	5.72E-04
PG(18:3_18:3)	PG	PL	4.84E-01	6.53E-02
PG(20:1_18:2)	PG	PL	-9.25E-01	7.51E-05
PI(14:0_18:2)	PI	PL	9.41E-01	1.93E-05
PI(14:0_18:3)	PI	PL	-9.88E-01	3.40E-08
PI(15:0_18:2)	PI	PL	-9.32E-01	3.14E-05
PI(15:0_18:3)	PI	PL	5.22E-01	6.95E-02
PI(16:0_18:2)	PI	PL	-8.87E-01	1.38E-04
PI(16:0_18:3)	PI	PL	8.89E-01	4.53E-04
PI(16:1_18:3)	PI	PL	9.39E-01	2.11E-05
PI(17:0_18:2)	PI	PL	9.51E-01	2.07E-05
PI(17:0_18:3)	PI	PL	9.59E-01	1.24E-05
PI(18:0_18:1)	PI	PL	9.74E-01	2.12E-06
PI(18:0_18:2)	PI	PL	9.54E-01	1.98E-05
PI(18:0_18:3)	PI	PL	9.83E-01	8.59E-07
PI(18:0_20:1)	PI	PL	8.87E-01	4.11E-04
PI(18:2_18:2)	PI	PL	9.68E-01	5.20E-06
PI(18:2_23:0)	PI	PL	-7.66E-01	4.52E-03
PI(18:2_23:1)	PI	PL	-7.87E-01	2.65E-03
PI(18:3_18:2)	PI	PL	7.96E-01	3.74E-03
PI(18:3_18:3)	PI	PL	9.94E-01	1.11E-08
PI(20:0_18:2)	PI	PL	9.03E-01	2.85E-04
PI(20:1_18:2)	PI	PL	5.85E-01	4.45E-02
PI(20:2_18:2)	PI	PL	-8.27E-01	9.43E-04
PI(20:3_18:2)	PI	PL	-6.58E-01	1.50E-02
PI(34:5)	PI	PL	-8.79E-01	2.71E-04
PI(34:6)	PI	PL	-2.88E-01	2.00E-01
PS(16:0_18:2)	PS	PL	-2.99E-01	1.82E-01
PS(16:0_18:3)	PS	PL	9.45E-01	3.16E-05
PS(18:0_18:2)	PS	PL	-2.27E-01	2.87E-01
PS(18:1_18:2)	PS	PL	2.61E-02	4.97E-01
PS(18:3_18:2)	PS	PL	7.53E-01	7.67E-03
PS(18:3_18:3)	PS	PL	9.79E-01	8.92E-07
PS(18:3_22:1)	PS	PL	8.89E-01	3.98E-04
PS(20:0_18:2)	PS	PL	-4.81E-01	7.25E-02
PS(20:1_18:3)	PS	PL	4.63E-01	1.01E-01
PS(22:0_18:2)	PS	PL	-1.86E-01	2.94E-01
PS(22:0_18:3)	PS	PL	1.03E-01	4.09E-01
PS(24:1_18:2)	PS	PL	2.25E-01	2.80E-01
PS(24:1_18:3)	PS	PL	8.66E-01	7.29E-04
So(d18:0)	So	So	-7.42E-01	8.11E-03
So(d18:0+pO)	So	So	-9.03E-01	2.33E-04
So(d18:1+hO)	So	So	-9.70E-01	2.05E-06
So(d20:0+pO)	So	So	-2.54E-01	2.58E-01
So(d22:0+pO)	So	So	-2.90E-01	2.23E-01
SQDG(16:0_18:3)	SQDG	GL	9.78E-01	6.47E-07
SQDG(18:2_18:3)	SQDG	GL	-6.39E-01	2.28E-02
StE(24:7)	StE	Sterols	-9.63E-01	3.32E-06
TG(10:0_10:1_18:1)	TG	TG	9.93E-01	9.44E-09
TG(10:0_18:2_18:3)	TG	TG	-8.65E-01	5.73E-04
TG(10:0_18:3_18:3)	TG	TG	5.19E-01	6.50E-02
TG(12:0_18:2_18:3)	TG	TG	-8.31E-01	1.40E-03
TG(12:0_18:3_18:3)	TG	TG	2.55E-01	2.43E-01
TG(14:0_18:2_18:3)	TG	TG	-8.27E-01	1.49E-03
TG(15:0_18:2_18:3)	TG	TG	-6.70E-01	1.64E-02
TG(15:0_18:3_18:3)	TG	TG	-5.66E-01	4.33E-02
TG(16:0_12:0_18:2)	TG	TG	-6.04E-01	3.06E-02
TG(16:0_14:0_18:2)	TG	TG	-7.89E-01	2.99E-03
TG(16:0_18:1_18:2)	TG	TG	-6.09E-01	2.89E-02
TG(16:0_18:2_18:3)	TG	TG	-8.59E-02	4.02E-01
TG(16:0_18:3_18:3)	TG	TG	4.55E-01	9.45E-02
TG(16:1_18:3_18:3)	TG	TG	-8.85E-01	3.23E-04
TG(17:0_18:3_18:3)	TG	TG	-3.04E-01	1.94E-01
TG(18:1_12:0_18:2)	TG	TG	-6.45E-01	2.13E-02
TG(18:1_18:2_18:3)	TG	TG	-6.35E-01	2.29E-02
TG(18:3_14:1_18:3)	TG	TG	-8.66E-01	5.96E-04
TG(18:3_17:1_18:3)	TG	TG	-9.01E-01	1.73E-04
TG(18:3_18:2_18:3)	TG	TG	-8.40E-01	1.13E-03
TG(18:3_18:2_20:3)	TG	TG	-5.43E-01	5.19E-02
TG(18:3_18:2_22:3)	TG	TG	-7.92E-01	3.05E-03
TG(18:3_18:3_18:3)	TG	TG	-8.36E-01	1.25E-03
TG(18:3_18:3_20:3)	TG	TG	7.21E-01	9.59E-03
TG(18:3_18:3_22:3)	TG	TG	7.94E-01	3.01E-03
TG(18:3_20:3_22:3)	TG	TG	6.44E-02	4.40E-01
TG(18:4_15:0_18:3)	TG	TG	1.28E-01	3.64E-01
TG(18:4_16:1_18:3)	TG	TG	-9.41E-01	2.42E-05
TG(18:4_17:1_18:3)	TG	TG	-9.29E-01	5.22E-05
TG(19:0_18:3_18:3)	TG	TG	-4.10E-01	1.15E-01
TG(19:1_18:3_18:3)	TG	TG	1.91E-01	3.04E-01
TG(20:1_18:3_18:3)	TG	TG	5.59E-01	4.77E-02
TG(4:0_16:0_18:3)	TG	TG	9.89E-01	6.73E-08
TG(43:9)	TG	TG	-9.31E-01	3.71E-05
WE(19:0_17:4)	WE	WE	-9.45E-01	1.16E-05
WE(3:0_18:1)	WE	WE	-9.83E-01	6.63E-08
WE(3:0_20:3)	WE	WE	-9.86E-01	6.88E-08
WE(3:0_20:4)	WE	WE	-9.80E-01	2.27E-07
WE(5:0_17:3)	WE	WE	1.15E-02	4.61E-01

Table S6 : List of p_{corr} from OPLS-DA and *P*-value from Welsch T-test of *Nicotiana sylvestris* seed samples CMSII vs WT

ID	family	cat	pcorr	pvalue
PI(18:1_18:2)	PI	PL	-0.996364	3.62E-20
PE(16:0_18:1)_11.38	PE	PL	-0.994034	2.38E-22
PE(16:0_18:1)_13.40	PE	PL	-0.994034	2.38E-22
PI(20:2_18:2)	PI	PL	-0.993624	1.79E-17
PG(16:0_17:0)	PG	PL	-0.992158	7.1E-20
PE(18:1_18:2)_10.29	PE	PL	-0.991432	3.52E-20
PE(18:1_18:2)_12.60	PE	PL	-0.991432	3.52E-20
PI(20:0_18:2)	PI	PL	-0.991039	1.49E-17
PC(16:0_18:1)	PC	PL	-0.990653	8.14E-20
PE(18:0_18:1)	PE	PL	-0.989073	3.08E-18
PI(18:2_18:2)	PI	PL	-0.98774	1.25E-15
PC(17:0_18:1)	PC	PL	-0.987249	2.69E-17
PE(20:0_18:2)_13.12	PE	PL	-0.986992	6.77E-17
PE(20:0_18:2)_14.89	PE	PL	-0.986992	6.77E-17
PI(16:0_18:1)	PI	PL	-0.986385	3.04E-15
PC(18:1_18:2)	PC	PL	-0.982547	6.68E-16
LPE(18:1)	LPE	PL	-0.981359	4.91E-15
MGDG(18:1_18:2)	MGDG	GL	-0.980285	1.77E-15
PC(16:1_18:3)	PC	PL	-0.980233	3.01E-15
PE(18:1_20:2)	PE	PL	-0.978868	6.82E-15
PI(18:0_18:1)	PI	PL	-0.9737	7.68E-13
PE(20:2_18:2)	PE	PL	-0.973586	8.21E-15
PC(17:1_18:2)	PC	PL	-0.96751	1.1E-11
LPE(16:0)	LPE	PL	-0.966928	1.27E-12
LPI(16:0)	LPI	PL	-0.963655	4.61E-11
PI(18:3_18:2)	PI	PL	-0.963501	1.21E-11
PI(17:0_18:1)	PI	PL	-0.963041	2.88E-11
MGDG(16:0_18:2)	MGDG	GL	-0.959608	2.69E-12
LPC(18:1)	LPC	PL	-0.956615	1.48E-10
LPC(16:0)	LPC	PL	-0.956551	1.47E-10
PG(16:0_16:1)	PG	PL	-0.948541	1.2E-10
PE(15:0_18:2)	PE	PL	-0.946606	6.01E-11
PC(18:0_18:1)	PC	PL	-0.945881	1.1E-10
PS(18:2_21:0)	PS	PL	-0.944763	1.68E-09
PC(20:1_18:2)	PC	PL	-0.941119	1.68E-10
LPC(16:1)	LPC	PL	-0.936103	2.98E-09
AcHexCmE(20:0)	AcHexCmE	Sterols	-0.935524	4.14E-09
LPC(20:2)	LPC	PL	-0.933888	2.87E-09
CL(74:5)	CL	PL	-0.930298	4.81E-09
PC(16:0_16:1)	PC	PL	-0.930034	1.83E-09
PC(17:1_16:0)	PC	PL	-0.919913	1.49E-09
PI(18:0_18:2)	PI	PL	-0.913975	4.29E-08
PS(20:0_18:2)	PS	PL	-0.912336	6.94E-08
SM(t38:2)	SM	SM	-0.909714	4.92E-08
PS(20:0_18:3)	PS	PL	-0.905216	1.14E-07
LPC(17:0)	LPC	PL	-0.901234	9.61E-08
DG(25:0_18:2)	DG	DG	-0.899892	1.3E-07
DG(16:0_18:1)	DG	DG	-0.898359	1.04E-07
PS(22:0_18:2)	PS	PL	-0.896809	2.75E-07
AcHexZyE(17:0)	AcHexZyE	Sterols	-0.892733	2.45E-07
PI(15:0_18:2)	PI	PL	-0.892632	2.06E-07
AcHexStE(18:0)	AcHexStE	Sterols	-0.892397	2.18E-07
LPI(18:2)	LPI	PL	-0.891187	3.25E-07
LPC(18:0)	LPC	PL	-0.889691	1.99E-07
LPC(18:2)	LPC	PL	-0.886421	2.46E-07
Hex1Cer(d42:3+O)	Hex1Cer	Cer	-0.883823	3.1E-07
AcHexStE(17:0)	AcHexStE	Sterols	-0.870549	9.1E-07
PI(16:0_18:3)	PI	PL	-0.865564	1.37E-06
PMe(16:0_18:1)	PMe	PL	-0.864535	1.25E-06
LPE(18:2)	LPE	PL	-0.861753	6.44E-07
MePC(38:1)_12.971	MePC	PL	-0.853082	2.13E-06
PS(36:3)	PS	PL	-0.85299	3.68E-06
DG(16:1_18:3)	DG	DG	-0.847399	2.85E-06
PS(18:0_18:2)	PS	PL	-0.844658	6.08E-06
SM(t34:2)	SM	SM	-0.830041	7.9E-06
AcHexSiE(17:0)	AcHexSiE	Sterols	-0.8287	9.7E-06
Cer(t40:2)	Cer	Cer	-0.818696	9.34E-06
PMe(18:1_18:2)	PMe	PL	-0.81869	1.19E-05
Hex1Cer(d34:3)	Hex1Cer	Cer	-0.818064	1.31E-05
PS(16:0_18:2)	PS	PL	-0.815784	1.97E-05
PI(16:0_16:0)	PI	PL	-0.807947	1.77E-05
Hex2Cer(d42:2+O)	Hex2Cer	Cer	-0.801831	2.48E-05
DG(18:0_18:1)	DG	DG	-0.796143	3.28E-05
DG(34:5)	DG	DG	-0.79001	3.46E-05
PMe(18:0_18:1)	PMe	PL	-0.781976	6.14E-05
DG(17:0_18:1)	DG	DG	-0.781219	5.63E-05
DG(17:1_16:0)	DG	DG	-0.78115	4.58E-05
Hex1Cer(d18:2_21:0+O)	Hex1Cer	Cer	-0.779218	5.69E-05
Hex1Cer(d18:2_25:0+O)	Hex1Cer	Cer	-0.77299	7.02E-05
DG(20:1_18:2)	DG	DG	-0.770431	8.13E-05
DG(18:1_18:2)_14.41	DG	DG	-0.764497	8.98E-05
PI(16:0_18:2)	PI	PL	-0.761912	0.00016
Hex1Cer(d18:2_15:0+O)	Hex1Cer	Cer	-0.74556	0.000174
TG(18:4_16:1_18:3)	TG	TG	-0.729513	0.000312
CL(72:4)	CL	PL	-0.726811	0.0003
Hex1Cer(d18:2_23:0+O)	Hex1Cer	Cer	-0.716019	0.000382
SPH(t18:1)	SPH	SPH	-0.71473	0.000355
PS(16:1_20:3)	PS	PL	-0.710599	0.000598
DG(20:1_18:1)	DG	DG	-0.70974	0.000498
AcHexSiE(18:0)	AcHexSiE	Sterols	-0.704235	0.000618
DG(42:2e)	DG	DG	-0.689342	0.000878
SM(t18:1_19:1)	SM	SM	-0.687375	0.000907
AcHexSiE(16:0)	AcHexSiE	Sterols	-0.658831	0.001625
PS(18:0_22:4)	PS	PL	-0.644109	0.002848
PE(16:0_18:3)	PE	PL	-0.633213	0.00303
CL(76:4)	CL	PL	-0.629394	0.002985
AcHexSiE(15:0)	AcHexSiE	Sterols	-0.595529	0.005947
DG(19:0_18:2)	DG	DG	-0.590104	0.00641
AcHexCmE(18:2)	AcHexCmE	Sterols	-0.589315	0.006677
DG(19:1_18:2)	DG	DG	-0.580824	0.006163
DG(16:0_16:0)	DG	DG	-0.574152	0.007023
PMe(20:2_18:2)	PMe	PL	-0.573359	0.008509
CL(70:1)	CL	PL	-0.558406	0.011433
DG(14:0_18:2)	DG	DG	-0.526216	0.015865
Hex1Cer(d18:2_22:0+O)	Hex1Cer	Cer	-0.525482	0.017915
CmE(20:4)	CmE	Sterols	-0.507338	0.022551
DG(18:2_21:0)	DG	DG	-0.488885	0.028694
TG(14:0_18:2_18:2)	TG	TG	-0.488244	0.032035
AEA(18:1)	AEA	AEA	-0.472299	0.036967
CL(70:3)	CL	PL	-0.465545	0.038543
AcHexStE(18:1)	AcHexStE	Sterols	-0.458098	0.043192
DG(17:1_18:2)	DG	DG	-0.456392	0.042645
DG(15:0_18:3)	DG	DG	-0.454396	0.043211
Hex1Cer(d18:2_24:0+O)	Hex1Cer	Cer	-0.422759	0.062443
Hex1Cer(t18:1_23:0+O)	Hex1Cer	Cer	-0.407636	0.074951
TG(12:0_18:2_18:2)	TG	TG	-0.390538	0.095864
TG(16:1_18:3_18:3)	TG	TG	-0.390447	0.092437
PMe(16:0_18:2)	PMe	PL	-0.373937	0.103238
CL(72:6)	CL	PL	-0.360714	0.116245
Cer(t17:1_21:0+O)	Cer	Cer	-0.334106	0.146517
Hex1Cer(d18:2_20:0+O)	Hex1Cer	Cer	-0.302133	0.192366
DG(33:0)	DG	DG	-0.279289	0.204662
DG(39:3e)	DG	DG	-0.276497	0.227424
PMe(18:2_18:2)	PMe	PL	-0.24749	0.282841
AcHexSiE(20:2)	AcHexSiE	Sterols	-0.244435	0.297474
DG(18:0_16:0)	DG	DG	-0.225279	0.301792
DG(15:0_18:2)	DG	DG	-0.18223	0.423259
PC(20:0_18:2)	PC	PL	-0.180241	0.395276
DG(20:2_18:2)	DG	DG	-0.1311	0.563772
DG(18:2_18:2)	DG	DG	-0.0739082	0.736049
DG(16:0_18:2)_15.16	DG	DG	-0.0325751	0.868578
MePC(34:5)	MePC	PL	0.00683912	0.995414
Hex1Cer(d18:2_26:0+O)	Hex1Cer	Cer	0.00764691	0.982836
DG(31:3e)	DG	DG	0.0484939	0.853009
TG(15:0_18:3_18:3)	TG	TG	0.0565007	0.808194
PE(14:0_18:2)	PE	PL	0.0636328	0.93321
Hex1Cer(d18:1_16:0+O)	Hex1Cer	Cer	0.0663336	0.795894
Hex2Cer(t41:0)	Hex2Cer	Cer	0.0791029	0.736032
TG(18:1_10:1_10:1)	TG	TG	0.0791808	0.761153
TG(16:0_18:3_18:3)	TG	TG	0.0982274	0.674028

TG(10:0_18:2_18:2)	TG	TG	0.211888	0.365136
PI(17:0_18:2)	PI	PL	0.251843	0.237561
CL(78:6)	CL	PL	0.260716	0.278081
Hex1Cer(d18:2_16:0)	Hex1Cer	Cer	0.291579	0.21457
CL(74:9)	CL	PL	0.296811	0.206055
Hex1Cer(t18:1_24:0+O)	Hex1Cer	Cer	0.317943	0.17872
Hex1Cer(t18:1_22:0+O)	Hex1Cer	Cer	0.318051	0.174493
LPC(24:0)	LPC	PL	0.335405	0.156656
PG(16:0_18:1)	PG	PL	0.362375	0.118446
ChE(20:0)	ChE	Sterols	0.381983	0.094397
SPH(t18:0)	SPH	SPH	0.395776	0.098388
Hex1Cer(t18:1_26:0+O)	Hex1Cer	Cer	0.416465	0.07132
LPC(22:0)	LPC	PL	0.43865	0.059181
DG(22:0_18:2)	DG	DG	0.465079	0.042046
DG(18:0_18:2)	DG	DG	0.466592	0.040752
DG(17:1_18:3)	DG	DG	0.488531	0.033332
DG(18:3_18:2)	DG	DG	0.528228	0.018864
DG(14:0_18:3)	DG	DG	0.545648	0.015543
DG(19:1_18:3)	DG	DG	0.612537	0.00473
Cer(t16:0_18:1)	Cer	Cer	0.657439	0.00222
PE(24:0_18:3)	PE	PL	0.663196	0.00121
PE(16:0_18:2)	PE	PL	0.673056	0.001598
DG(17:0_18:2)	DG	DG	0.676388	0.0013
PE(18:3_18:3)	PE	PL	0.71774	0.000495
TG(18:2_10:1_10:1)	TG	TG	0.755582	0.000159
DG(22:2_18:2)	DG	DG	0.76522	0.000117
TG(19:1_18:2_18:3)	TG	TG	0.766583	9.4E-05
AcHexSiE(20:0)	AcHexSiE	Sterols	0.772029	8.55E-05
CL(82:6)	CL	PL	0.776125	7.86E-05
DG(18:3_18:3)	DG	DG	0.776271	8.11E-05
TG(18:3_18:2_18:3)	TG	TG	0.7907	3.66E-05
TG(18:3_10:1_10:1)	TG	TG	0.81561	1.87E-05
PG(18:0_18:1)	PG	PL	0.840787	3.27E-06
Hex1Cer(d18:2_18:0)	Hex1Cer	Cer	0.854257	3.04E-06
DG(41:5e)	DG	DG	0.856103	2.08E-06
MGDG(18:3_18:3)	MGDG	GL	0.863149	7.81E-07
TG(19:1_18:3_18:3)	TG	TG	0.878311	4.54E-07
PG(16:0_16:0)	PG	PL	0.892081	1.14E-07
TG(18:3_17:1_18:3)	TG	TG	0.892174	1.69E-07
DG(18:3_22:2)	DG	DG	0.895797	1.94E-07
PC(19:0_18:2)	PC	PL	0.896794	6.34E-08
PG(18:1_18:2)	PG	PL	0.914099	1.73E-08
PC(16:0_18:3)	PC	PL	0.921893	1.55E-09
PG(18:0_16:0)	PG	PL	0.925176	4.9E-09
Cer(t17:1_22:0+O)	Cer	Cer	0.929561	1.64E-09
Cer(d34:0+O)	Cer	Cer	0.932114	2.65E-09
Cer(t18:0_16:0)	Cer	Cer	0.932114	2.65E-09
PE(18:2_18:2)	PE	PL	0.933781	3.56E-09
Cer(t17:1_24:0+O)	Cer	Cer	0.93451	1.68E-09
PC(15:0_18:2)	PC	PL	0.937231	2.62E-09
Cer(t18:0_20:0)	Cer	Cer	0.942159	1.83E-10
TG(18:3_18:3_18:3)	TG	TG	0.946114	8.07E-10
PC(16:0_16:0)	PC	PL	0.949636	3.67E-10
PE(18:0_18:2)	PE	PL	0.953264	8.12E-10
PC(18:0_16:0)	PC	PL	0.955167	1.29E-10
PE(18:3_18:2)	PE	PL	0.960422	5.84E-11
Cer(t17:1_23:0+O)	Cer	Cer	0.961162	1.09E-11
Cer(d18:2_16:0+O)	Cer	Cer	0.966188	2.15E-11
PE(18:1_22:0)	PE	PL	0.969088	3.72E-12
MePC(41:5)	MePC	PL	0.974373	4.92E-12
MGDG(18:2_18:2)	MGDG	GL	0.979972	1.47E-13
PC(18:2_18:2)	PC	PL	0.980402	1.21E-14
Cer(t44:1+O)	Cer	Cer	0.981062	1.06E-14
Cer(t18:0_24:0)	Cer	Cer	0.981169	4.25E-15
Cer(t17:1_25:0+O)	Cer	Cer	0.981941	4.26E-15
Cer(t18:0_21:0)	Cer	Cer	0.982218	1.02E-15
Cer(t18:0_23:0)	Cer	Cer	0.983275	7.58E-16
PC(16:0_18:2)	PC	PL	0.983955	3.48E-14
PC(20:2_18:2)	PC	PL	0.983959	5.23E-14
Cer(t18:0_22:0)	Cer	Cer	0.984241	5.11E-16
PC(18:3_18:3)	PC	PL	0.984762	4.71E-15
PG(18:2_18:2)	PG	PL	0.98504	1.2E-15
PE(22:0_18:2)	PE	PL	0.98826	4.27E-16

PG(18:3_18:2)	PG	PL	0.990535	4.73E-17
PG(16:0_18:3)	PG	PL	0.990624	1.44E-17
PE(24:0_18:2)	PE	PL	0.990903	4.98E-17
dMePE(20:2_18:2)	dMePE	PL	0.991321	3.83E-17
PE(22:2_18:2)	PE	PL	0.991321	3.83E-17
PG(16:0_18:2)	PG	PL	0.99359	1.42E-20
PC(18:3_18:2)	PC	PL	0.99539	1.39E-20
PC(24:0_18:2)	PC	PL	0.995712	1.11E-20
PC(22:2_18:2)	PC	PL	0.995846	4.38E-21
PC(17:0_18:2)	PC	PL	0.996529	3.81E-22
PC(18:0_18:2)	PC	PL	0.99655	5.52E-21
PC(22:0_18:2)	PC	PL	0.997316	4.29E-22

Table S7 List of p_{corr} from OPLS-DA and *P*-value from Welsch T-test of *Nicotiana sylvestris* pollen samples CMSII vs WT

Primary ID	family	cat	pcorr	pvalue
cPA(18:2)	cPA	PL	-0.99888	1.55E-06
TG(18:3_18:3_18:3)	TG	TG	-0.99775	9.36E-08
TG(25:0_18:3_18:3)	TG	TG	-0.99745	2.50E-06
DG(18:1_18:1)	DG	DG	-0.99744	3.41E-06
PC(40:3)	PC	PL	-0.99626	1.40E-08
DG(17:1_18:3)	DG	DG	-0.9958	2.10E-05
TG(22:0_18:2_18:3)	TG	TG	-0.9958	1.08E-08
PC(38:1)	PC	PL	-0.99564	1.11E-06
TG(24:0_18:3_18:3)	TG	TG	-0.99545	1.00E-05
DG(18:1_18:2)	DG	DG	-0.99539	1.27E-05
TG(24:0_18:2_18:3)	TG	TG	-0.99538	5.36E-07
DG(18:3_18:3)	DG	DG	-0.99524	2.31E-05
TG(17:0_18:3_18:3)	TG	TG	-0.99502	2.14E-05
TG(16:1_18:2_18:3)	TG	TG	-0.99492	6.78E-09
TG(18:3_18:2_21:0)	TG	TG	-0.99425	8.86E-07
cPA(18:3)	cPA	PL	-0.99362	8.25E-06
TG(25:0_18:2_18:3)	TG	TG	-0.99348	1.63E-05
Cer(d18:2_16:0+O)	Cer	Cer	-0.99301	1.28E-05
PC(42:3)	PC	PL	-0.9923	1.11E-05
DG(18:2_23:0)	DG	DG	-0.99194	3.51E-05
DG(16:0_16:0)	DG	DG	-0.9913	3.85E-06
DG(18:3_23:0)	DG	DG	-0.99077	6.27E-05
TG(24:0_18:2_18:2)	TG	TG	-0.99021	1.69E-07
TG(16:0_18:2_22:0)	TG	TG	-0.99019	1.33E-06
TG(17:0_18:2_18:3)	TG	TG	-0.99019	3.93E-05
PG(18:1_18:2)	PG	PL	-0.98946	3.11E-05
TG(18:4_17:1_18:3)	TG	TG	-0.98919	7.32E-05
DG(15:0_18:2)	DG	DG	-0.98814	7.20E-07
DG(16:0_16:1)	DG	DG	-0.98808	2.50E-07
PC(38:0)	PC	PL	-0.98678	1.29E-06
TG(22:2_18:2_18:2)	TG	TG	-0.98624	1.85E-07
PE(24:0_18:2)	PE	PL	-0.98574	6.63E-07
PC(36:1)	PC	PL	-0.9856	0.000111
PC(44:2)	PC	PL	-0.9848	0.000116
LPC(18:1)	LPC	PL	-0.98441	5.79E-05
TG(22:0_18:2_18:2)	TG	TG	-0.98415	1.64E-07
PE(25:0_18:2)	PE	PL	-0.98398	1.24E-06
TG(18:1_18:2_21:0)	TG	TG	-0.98392	9.83E-05
DG(18:2_18:2)	DG	DG	-0.98347	0.000192
PG(18:3_18:2)	PG	PL	-0.98313	8.32E-05
TG(16:1_18:3_18:3)	TG	TG	-0.98126	0.000196
PE(26:0_18:3)	PE	PL	-0.98086	1.66E-06
CerG1(d18:2_26:0+O)	CerG1	Cer	-0.98067	0.000215
TG(18:3_17:1_18:3)	TG	TG	-0.97808	0.000231
TG(20:1_18:1_18:2)	TG	TG	-0.9771	6.65E-06
DG(14:0_18:3)	DG	DG	-0.97664	1.26E-06
PC(40:2)	PC	PL	-0.97634	0.000314
CerG1(d18:2_17:0+O)	CerG1	Cer	-0.97052	2.95E-06
LPC(18:2)	LPC	PL	-0.96355	0.000655
LPC(16:1)	LPC	PL	-0.96346	0.000497
TG(18:1_18:2_24:0)	TG	TG	-0.95985	0.000264
DG(17:0_18:3)	DG	DG	-0.95904	2.71E-05
LPC(15:0)	LPC	PL	-0.95563	0.000844
TG(26:0_16:0_18:2)	TG	TG	-0.94744	0.000888
PC(36:2)	PC	PL	-0.94432	0.000338
PC(33:3)	PC	PL	-0.92453	0.000894
TG(22:0_18:2_22:0)	TG	TG	-0.89117	0.004361
PC(32:1)	PC	PL	-0.88623	0.003511
LPC(17:0)	LPC	PL	-0.8714	0.001216
TG(18:3_18:2_18:2)	TG	TG	-0.86855	0.001386
CerG1(d18:2_16:0+O)	CerG1	Cer	-0.86575	0.001081
PC(33:4)	PC	PL	-0.81559	0.004614
PE(16:0_16:0)	PE	PL	-0.64633	0.043411
PE(18:0_18:1)	PE	PL	-0.59372	0.078046
LPC(16:0)	LPC	PL	-0.45529	0.209559
PE(17:0_18:3)	PE	PL	-0.1558	0.675082
Cer(d36:3)	Cer	Cer	0.199213	0.600423
ChE(18:3)	ChE	Sterols	0.554787	0.126033
PI(18:3_18:3)	PI	PL	0.623243	0.058693
TG(18:0_18:0_18:0)	TG	TG	0.774719	0.02645
TG(18:3_18:2_20:3)	TG	TG	0.800346	0.016719
DG(20:2_18:2)	DG	DG	0.819169	0.003636
PC(36:6p)	PC	PL	0.908296	0.001053
TG(18:3_18:3_22:3)	TG	TG	0.908535	0.001841
TG(18:3_18:2_20:2)	TG	TG	0.917667	0.002114
AcCa(18:3)	AcCa	AcCa	0.924536	0.00021
PC(32:2)	PC	PL	0.924793	0.000915
PI(18:3_18:2)	PI	PL	0.938754	0.00016
WE(5:0_26:1)	WE	WE	0.939134	0.000302
WE(3:0_20:2)	WE	WE	0.940534	0.00052
SQDG(43:6)	SQDG	GL	0.946801	0.000762
TG(18:0_18:2_18:3)	TG	TG	0.953322	0.000173
PC(31:0)	PC	PL	0.958304	2.26E-05
TG(18:3_18:2_22:3)	TG	TG	0.960741	0.000245
TG(4:0_18:1_24:0)	TG	TG	0.961916	0.000505
TG(22:3_18:2_18:2)	TG	TG	0.962445	0.000397
WE(5:0_24:0)	WE	WE	0.96656	0.000112
WE(5:0_26:2)	WE	WE	0.971626	0.000123
TG(24:1_18:3_18:3)	TG	TG	0.972573	4.96E-06
TG(45:3e)	TG	TG	0.980581	6.22E-05
PC(33:2)	PC	PL	0.980973	5.86E-07
PG(18:2_18:2)	PG	PL	0.984116	2.11E-07
PC(32:0)	PC	PL	0.984612	2.71E-07
TG(4:0_18:2_22:0)	TG	TG	0.985399	6.39E-05
TG(6:0_16:1_22:1)	TG	TG	0.985411	6.37E-05
MG(26:0)	MG	PL	0.988323	2.05E-05
cPA(16:0)	cPA	PL	0.989615	6.41E-08
PC(34:0)	PC	PL	0.993883	2.76E-06
PC(33:1)	PC	PL	0.995444	1.01E-07
